# Lipid based nanoparticles as a novel treatment modality for hepatocellular carcinoma: a comprehensive review on targeting and recent advances

**DOI:** 10.1186/s12951-022-01309-9

**Published:** 2022-03-05

**Authors:** Khaled Mahmoud, Shady Swidan, Mohamed El-Nabarawi, Mahmoud Teaima

**Affiliations:** 1 Department of Pharmaceutics and Pharmaceutical Technology, Faculty of Pharmacy, The British University in Egypt, El-Sherouk City, Cairo, 11837 Egypt; 2 The Center for Drug Research and Development (CDRD), Faculty of Pharmacy, The British University in Egypt, El-Sherouk City, Cairo, 11837 Egypt; 3 Department of Pharmaceutics and Industrial Pharmacy, Faculty of Pharmacy, Cairo University, Cairo, 11562 Egypt

**Keywords:** Lipidic nanoparticles, Targeting approaches, Liposomes, Solid lipid nanoparticles, Nanostructured lipid carriers

## Abstract

Liver cancer is considered one of the deadliest diseases with one of the highest disease burdens worldwide. Among the different types of liver cancer, hepatocellular carcinoma is considered to be the most common type. Multiple conventional approaches are being used in treating hepatocellular carcinoma. Focusing on drug treatment, regular agents in conventional forms fail to achieve the intended clinical outcomes. In order to improve the treatment outcomes, utilizing nanoparticles—specifically lipid based nanoparticles—are considered to be one of the most promising approaches being set in motion. Multiple forms of lipid based nanoparticles exist including liposomes, solid lipid nanoparticles, nanostructured lipid carriers, microemulsion, nanoemulsion, phytosomes, lipid coated nanoparticles, and nanoassemblies. Multiple approaches are used to enhance the tumor uptake as well tumor specificity such as intratumoral injection, passive targeting, active targeting, and stimuli responsive nanoparticles. In this review, the effect of utilizing lipidic nanoparticles is being discussed as well as the different tumor uptake enhancement techniques used.

**Graphical Abstract:**

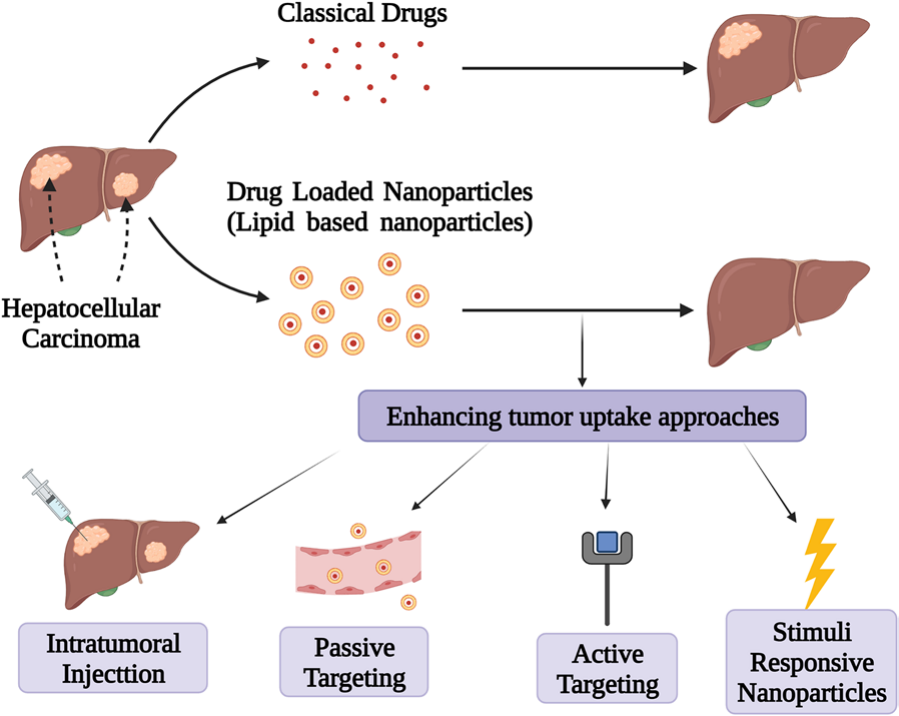

## Introduction

**Fig. 1:**
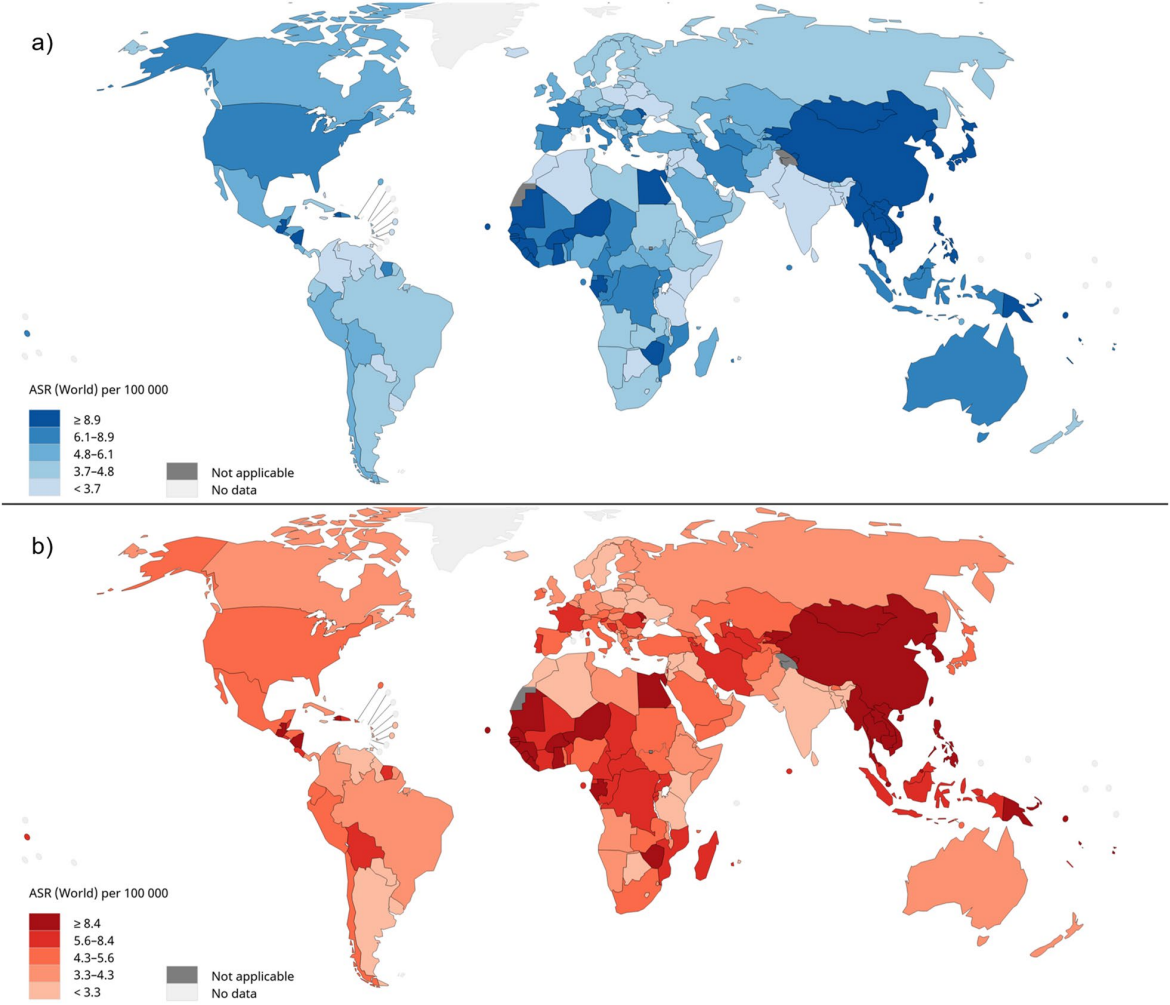
Global age-adjusted **a** incidence rates, **b** mortality rates of liver cancer, estimated for 2020. Data source: GLOBOCAN 2020. Graph production: IARC (http://gco.iarc.fr/today), World Health Organization

Liver cancer is the sixth highest cancer in terms of incidence rate and the third highest cancer in mortality rates. The world health organization (WHO) estimates the occurrence of 905,677 new cases in the year 2020 with 830,180 new deaths during the same year (Fig. 1). Males are a higher risk group for liver cancer. Eastern Asia, northern Africa, and Micronesia are the three highest regions in terms of incidence rates. However, eastern Asia, northern Africa, and south-eastern Asia are the three highest regions in terms of mortality rates [1, 2]. Hepatocellular carcinoma (HCC) accounts for more than 80% of all primary liver cancer cases [3]. Making it the most important type to be focused on.

Development of HCC is often associated with the induction of inflammation related hepatic injury. Chronic inflammation results in necrotic effect in hepatocytes which triggers a regeneration process. This process results in chronic liver disease which leads to the generation of fibrosis followed by cirrhosis [4]. Cirrhosis is the most common predisposing factor for HCC development [5]. Excessive metabolic and oxidative stress in cirrhotic liver results in excessive hepatocytes regeneration. High hepatocytes turnover results in the accumulation of genetic errors and mutations resulting in the formation of dysplastic hepatocytes and hepatic nodules resulting in HCC occurrence [6]. HCC can occur as well -yet less commonly- in non-cirrhotic liver [7]. Factors such as hepatitis viruses, carcinogens, and fatty liver diseases results in the suppression of the tumor suppressor p53, oxidative stress, and inflammation. These effects result in genetic mutations and the activation of oncogenic pathways [8]. Multiple genes have been identified to be altered in case of HCC including TP53, retinoblastoma RB1, CDKN2A, insulin-like growth factor-2 receptor, and CTNNBI (β-catenin) [9].

Several etiological risk factors have been related to HCC which directly influence disease progression [10]. These risk factors include chronic infection with hepatitis B and C viruses, alcoholic liver disease, nonalcoholic steatohepatitis, obesity, diabetes, intake of food contaminated with aflatoxin and hemochromatosis [11].

**Fig. 2:**
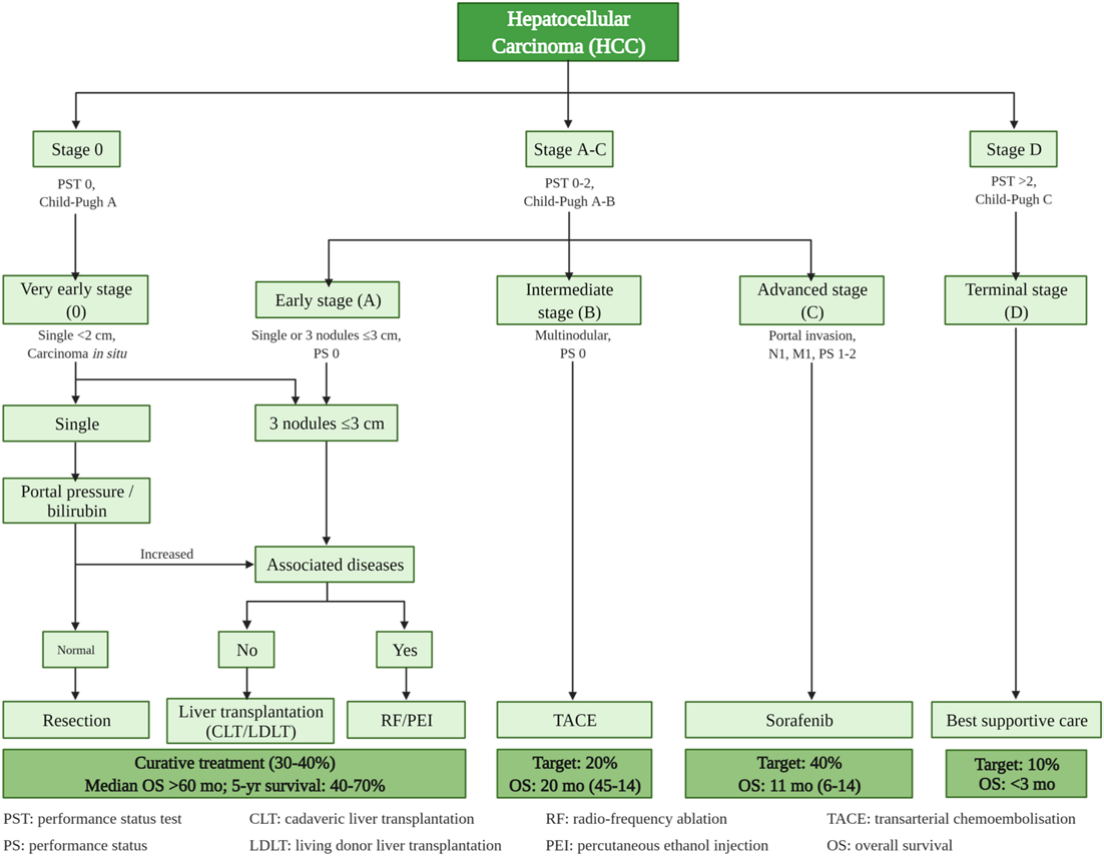
BCLC Staging system and the associated treatment algorithm (Adopted from reference [14] with permission. Copyright 2012, Elsevier. Created with BioRender.com)

Varying staging systems have been employed to classify HCC such as Okuda, CLIP (Cancer of the Liver Italian Program) score, and HKLC (Hong Kong Liver Cancer) staging [12]. However, the most widely used staging system is the Barcelona Clinic Liver Cancer (BCLC) staging being recommended by the European Association for the Study of the Liver (EASL) and the American Association for the Study of Liver Diseases (AASLD) [13]. BCLC (Fig. 2) [14] classify HCC into 5 stages -0 (very early stage), A (early stage), B (intermediate stage), C (advanced stage), and D (terminal stage)-depending on multiple variables including tumor status, liver functional status, physical status, and cancer-related symptoms. These stages are linked to a treatment algorithm [15].

Other classification systems have been employed based on the molecular signature of HCC. These classification systems include G1-G6 (gene-signature based classification), iHCC 1–3 (metabolic classification), immune-high, immune-mid and immune-low (immunological classification) and poorly polyploid and highly polyploid (chromosomal classification) [16].

Current conventional HCC therapies include liver resection, liver transplantation, local ablative therapy, transarterial therapy, and systemic therapies (tyrosine kinase inhibitors). However, regular chemotherapeutic agents such as doxorubicin (DOX) and gemcitabine show limited success due to the high ability of HCC to possess drug resistance both intrinsic and acquired [17].

Liver resection is the process by which part of liver is removed. The extend of partial removal is governed by the tumor extension as well as the histological change of the underlying parenchyma. However, liver transplantation possesses the ability to completely remove both detectable, un-detectable nodules, preneoplastic lesions as well as treating the underlying cirrhosis [18]. Choosing between liver resection or liver transplantation within the Milan criteria while possessing good liver function is still unclear due to the limitations of both techniques. Liver transplantation holds high surgical morbidity and mortality rates while liver resection results in poor long-term benefits due to high risk of recurrence and low disease-free survival rates [19].

Local ablative therapy is considered to be the go-to therapy for early stage, unresectable tumors [20]. Locally applied ablation possesses the advantages of low impact on the body as a result of targeting only the tumor and its surrounding tissue, high efficiency and short application time [21]. Local ablation can be achieved through various techniques including the application of chemical agents such as ethanol and acetic acid as well as the application of an energy source including thermal ablation (radiofrequency ablation, microwave ablation, and laser photocoagulation), cryoablation, high-intensity focused ultrasound, and irreversible electroporation [22]. However, applying thermal ablation is contraindicated in certain cases including extrahepatic disease, tumor adjacent to a major hepatic duct, the presence of lesions larger than 5 cm, and the existence of more than four lesions [23].

Transarterial therapy can be classified into transarterial embolization, transarterial chemoembolization (TACE), and transarterial radioembolization (TARE) [24]. Transarterial embolization is a technique that depends on blocking the main artery supplying the tumor tissue with its needed nutrients through injecting an embolic agent. This results in ischemic necrosis of the intended tumor [25]. TACE is done through injecting a chemotherapeutic agent into the artery followed by transarterial embolization [26]. TARE depends on injecting microspheres saturated with a radioactive isotope (yttrium-90) [27].

Genetic mutations in cancer setting result in dysregulating tyrosine kinases [28]. Tyrosine kinases include multiple important proteins in HCC that exert important roles in HCC pathogenesis including vascular endothelial growth factor receptor (VEGFR) [29]. Sorafenib (SOR) was the first approved tyrosine kinase inhibitor. It significantly prolonged progression time as well as overall survival time [30]. A total of six tyrosine kinases have been approved for HCC treatment. Lenvatinib and donafenib alongside SOR are considered to be first line therapy. Regorafenib, cabozantinib, and apatinib are considered to be second line treatments. Other treatments other than tyrosine kinase inhibitors include bevacizumab, nivolumab, and ramucirumab [31].

Despite multiple approved drugs, drug treatment of unresectable, advanced HCC still does not meet the intended outcomes. These insufficient outcomes can be attributed to multiple reasons including low drug bioavailability, and nonspecific drug delivery which lead to high risk of side effects with low drug concentration in the target tissue [32]. One of the important approaches used to increase treatment efficacy is the utilization of nanoparticles [33]. Other approaches include the development of new systemic therapies [34]. However, the development process of new systemic drugs is slow as it took almost 10 years to expand the variety of medications to incorporate drugs other than SOR [35].

Nanoparticles are defined recently by the British Standards Institution as 3D nano-objects with 3 external dimensions in the nano range with the nano range extending between 1 and 1000 nm [36]. Nanoparticles possess multiple advantages such as payload stability, tumor specific delivery, high intracellular uptake, high surface-to-volume ratio, ability to co-encapsulate multiple therapeutic agents [37] as well as the ability to enhance bioavailability [38]. Multiple types of nanoparticles exist which include polymeric nanoparticles, lipidic nanoparticles, metallic nanoparticles, and silica-based nanoparticles. However, the majority of clinically approved drug delivery nanoparticles are either polymeric or lipidic in nature [39]. Lipidic nanoparticles possess huge potential in treating liver diseases in general. This potential is manifested in the drug ONPATTRO® which is the first clinically approved liver targeting nanomedicine [40]

Lipidic nanoparticles are biocompatible, non-toxic, and well tolerated nanoparticles formulated mainly of physiological lipids [41]. Using lipidic nanoparticles is advantageous in providing enhanced physical stability, lower toxicity due to the absence of organic solvents, ease of scalability, and relatively low production cost [42].

**Fig. 3:**
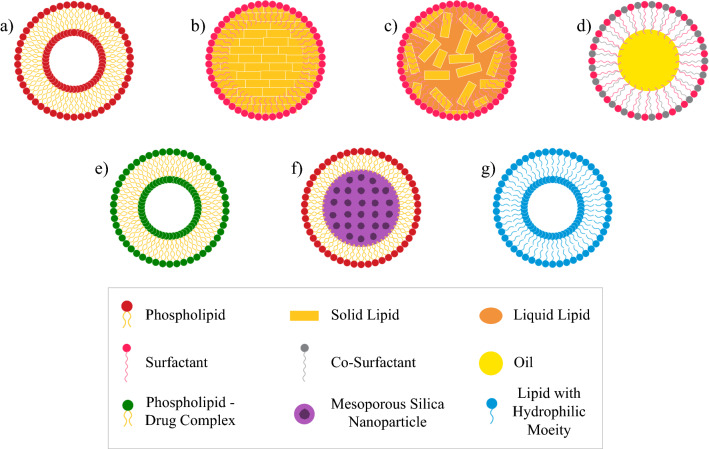
Schematic presentation of lipidic nanoparticles; **a** Liposomes, **b** Solid Lipid Nanoparticles, **c** Nanostructured Lipid Carriers, **d** Micro/Nano emulsion, **e** Phytosomes, **f** Lipid Coated Nanoparticles, **g** Nanoassemblies

This review article is constructed with the aim of elucidating the huge potential of using lipidic nanoparticles (Fig. 3) to deliver anti-neoplastic drugs for HCC treatment. Through analyzing a decade long of research, the positive impacts on encapsulating anti-neoplastic drugs with various lipidic nanoparticles are exhibited. Various targeting approaches and their impact are also demonstrated. Other review articles discussed different nanoparticles in HCC [43, 44] as well as nanoparticles targeting in HCC [45, 46]. However, to the best of our knowledge, this is the first review article to focus on lipidic nanoparticles in delivering anti-neoplastic drugs for HCC.

## Lipidic nanoparticles in HCC treatment

Lipidic nanoparticles possess the ability to improve the encapsulated drugs bioavailability and permeability, as well as enhance the payload stability through providing protection against physiological barriers [47]. These effects are prominent in the case of insoluble active pharmaceutical ingredients with low stability, and low bioavailability resulting in better drug pharmacokinetics [48]. Different lipidic nanoparticles possess varying characteristic advantages and limitations rationing the use of each type of these nanoparticles. Different advantages and limitations of each type of lipidic nanoparticles are summarized in Table 1.

**Table 1:** Advantages and limitations of lipid based nanoparticles

Nanoparticles	Advantages	Limitations
Liposomes	Biocompatible Biodegradable Non-immunogenic Low toxicity [301]	High Production Cost Drug leakage Short half-life Possible oxidation and hydrolysis of the used phospholipid [302]
Solid Lipid Nanoparticles (SLNs)	Biocompatible Does not involve the use of organic solvents (green synthesis) Reproducible and scalable manufacturing process [303]	Low encapsulation efficiency as a result of perfect crystalline structure High drug expulsion [304]
Nanostructured Lipid Carriers (NLCs)	High encapsulation efficiency Low drug expulsion [99]	Possible cytotoxic effect depending on the matrix structure Irritating action of some surfactants [305]
Micro/ Nano emulsion	Self-assembly High penetration through the biological membranes [306] High absorption rate [307]	High concentrations of surfactants Possible phase separation [306]
Phytosomes	Enhanced absorption Low toxicity [308]	Phytochemical leaching Low drug concentration [309]
Lipid Coated Nanoparticles	Biocompatibility Structural stability Flexibility in conjugating targeting moieties [310]	Multi-step fabrication process Challenging scale-up [311]
Nanoassemblies	Rapid synthesis Easy dispersibility Low production cost [312]	Difficult to control particle size Challenging scale-up Low shelf-life stability [312]

### Liposomes

Liposomes are considered to be one of the most studied nanoparticles [49]. Liposomes are vesicular systems composed of one or more phospholipid bilayer surrounding an inner aqueous space [50]. They have the ability to entrap hydrophobic drugs in the bilayer area as well as hydrophilic drugs in the inner aqueous space [51].

Liposomes have been extensively studied as an approach to HCC treatment. Yang et al. prepared liposomal formulation entrapping docetaxel. The prepared liposomes composed of soy phosphatidylcholine, 1,2-dimyristoyl-sn-glycero-3-phosphocholine (DMPC), or 1,2-dioleoyl-sn-glycero-3-phosphocholine (DOPC). HepG2 and SMMC-7721 cells were used to assess the efficacy of drug encapsulation in liposomal formulation as well as the safety of different liposomes forming materials. In both cells, soy phosphatidylcholine was the safest liposome forming material at both 24 and 48 h except for SMMC-7721 cells at 48 h, DMPC was the safest option. In HepG2 cells, drug loaded DOPC liposomes exhibited the highest cytotoxicity. While in SMMC-7721, drug loaded soy phosphatidylcholine liposomes had the highest cytotoxicity. It is also worth mentioning that in both cell lines, combining free drug with blank liposomes demonstrated higher cytotoxicity than drug encapsulated liposomes. Drug loaded soy phosphatidylcholine liposomes were able to decrease lactate dehydrogenase leakage, alleviate drug induced intracellular malondialdehyde (MDA) production, while increasing superoxide dismutase activity compared to free drug in HepG2 cells [52].

Chitosan can be applied as a coat to increase the liposomes stability and minimize its aggregation [53]. Quagliariello et al. prepared chitosan coated liposomes containing butyric acid. The prepared liposomes composed of sodium phosphatidylcholines and cholesterol. Applying chitosan coating didn’t affect the cytotoxicity with minimal toxicity induced by the liposomal formulation indicating the safety of the preparation when assessing the cellular viability in the absence of the drug. The prepared drug loaded liposomes both coated and non-coated exhibited higher cytotoxic effects against HepG2 cells compared to free drug with coated liposomes being the most effective. Chitosan coated drug loaded liposomes had the highest uptake following clathrin-dependent endocytosis. Chitosan coated drug loaded liposomes were also able to induce significantly higher anti-inflammatory effects in HepG2 cells compared to free drug [54]. Erythrocyte membrane is another coat that can be applied to the surface of nanoparticles [55]. AlQahtani et al. entrapped 5-fluorouracil in biomimetic liposomes coated with nanoerythrocyte membrane. The prepared liposomes composed of 1,2-dipalmitoyl-sn-glycero-3-phosphocholine (DPPC) and cholesterol. When assessing the cytotoxicity of the prepared nanoparticles in HepG2 cells, the prepared drug loaded nanoparticles exhibited the least cytotoxic effect compared to free drug, drug loaded liposomes, and drug loaded nanoerythrocytes. The authors attributed these results to the sustained release effect of the prepared drug loaded nanoparticles delaying its cytotoxic activities. The prepared blank nanoparticles exhibited negligible cytotoxicity indicating its safety [56]. The research group expanded their work through comparing drug loaded chitosan coated poly lactic-co-glycolic acid (PLGA) nanoparticles, chitosan coated liposomes, nanoerythrocytes, nanoerythrocytes coated chitosan coated PLGA nanoparticles, and nanoerythrocytes coated chitosan coated liposomes. After 72 h, nanoerythrocytes coated chitosan coated PLGA nanoparticles exhibited high cell attraction and high targeting capability in HepG2 cells. In vivo testing in healthy rats exhibited the ability of nanoerythrocytes coated chitosan coated PLGA nanoparticles to enhance the drug pharmacokinetic parameters including larger AUC and longer t_1/2_ compared to chitosan coated PLGA nanoparticles and free drug. Nanoerythrocytes coated chitosan coated PLGA nanoparticles also had higher drug accumulation in the liver [57].

Due to conventional chemotherapeutics agents’ drawbacks such as toxicity and cancer recurrence, there is an ongoing search for new compounds with anti-cancer activity. One of the most explored options is the usage of phytochemicals [58]. Phytochemicals are defined as non-nutrient, secondary metabolite, plant derived compounds that exert biological actions on the human body [59]. Multiple phytochemicals have been explored in the treatment of HCC using liposomes. Jain et al. explored the efficacy of green tea catechins encapsulated in liposomal formulation. The authors different kinds of liposomes using L-a-phosphatidylcholine, cholesterol, dicetyl phosphate, stearylamine, and 1,2-distearoyl-sn-glycero-3-phosphoethanolamine (DSPE)-mPolyethyle Glycol (PEG) 2000. The authors chose the liposomes, anionic liposomes, and pegylated liposomes for further evaluation. In vitro evaluation using HepG2 cells showed higher cytotoxic effect for anionic liposomes when compared to other liposomal formulation and free catechins after 72 h. It also showed higher apoptosis for anionic liposomes in comparison to free catechins and control. Ehrlich ascites carcinoma model showed anionic liposomes induced longer survival time, enhanced control over hematological parameters, and improved endogenous antioxidant activity [60]. Jagwani et al. formulated cationic liposomes formulated from soy lecithin, cholesterol, stearylamine that was used to entrap resveratrol. Cationic liposomes showed higher uptake in HepG2 cells, lower cell viability as well as lower IC_50_ compared to blank nanoparticles and free drug. Pharmacokinetic testing demonstrated that drug loaded liposomes were able to accumulate more in the plasma and liver compared to free drug while the free drug and drug loaded liposomes were both not toxic to the N-nitrosodiethylamine rats’ vital organs. Drug loaded liposomes therapeutic and protective effects were assessed in which the prepared liposomes exhibited the highest therapeutic and cancer preventive effect in comparison to free drug. Resveratrol loaded cationic liposomes also showed lower number of nodules as well as lower liver marker enzymes [61].

Combination therapy has been extensively used in cancer therapy. Combination therapy utilizes the pharmacological actions of two or more pharmaceutical agents in either an additive or a synergistic manner in order to combat the heterogenic nature of cancers [62]. Employing combination therapy in cancer treatment has shown great success in increasing treatment efficacy, reversing multidrug resistance (MDR), as well as reducing in vivo toxicity [63]. Yin et al. formulated liposomes entrapping ceramide alongside SOR, the liposomes consisted of Lipoid® E80, DSPE-methoxyPEG(mPEG)2000, and cholesterol. The formulation was successfully uptaken by HepG2 calls. The prepared liposomes showed synergistic cytotoxic effect on HepG2 when compared to single drug liposomes. However, free SOR and free ceramide showed the lowest cell viability and the lowest IC_50_ respectively, which the authors attributed to the easier availability of the drugs to the cancerous cells. Yet, the authors also stated that these results may indicate longer action time of the prepared liposomes resulting in higher anti-proliferative effect. In vivo testing was done on H22 tumor bearing mice on which the prepared formulation showed the lowest tumor volume as well as the lowest tumor weight, while decreasing SOR exposure to normal tissue [64]. Wang et al. prepared liposomes composed of hydrogenated soy phosphatidylcholine (HSPC) and cholesterol that were used to entrap DOX and lovastatin. In vivo pharmacokinetic study showed that combination containing liposomes showed higher AUC and lower clearance rate comparing to DOX containing liposomes indicating the positive effect of lovastatin in enhancing the bioavailability of DOX. H22 mice model showed that the combination liposomes had the lowest tumor volume, lowest tumor weight, highest survival rate while attenuating the toxic effects of DOX. The prepared liposomes also showed higher accumulation of the prepared liposomes in the tumor tissue [65]. Sarfraz et al. also used DOX which was entrapped with oleanolic acid in liposomal formulation composed of HSPC, cholesterol, and DSPE-PEG2000. Combination loaded liposomes had the lowest cell viability percentage on HepG2 cells. HepG2 tumor-bearing nude mice showed that free DOX was more effective in inhibiting the tumor growth as well as reducing tumor weight. However, free DOX was found to be more toxic to other organs as well. The authors stated that the combination loaded liposomes were able to decrease DOX related toxicity through conducting a toxicity study using H9C2 cardiomyocytes as well as measuring various markers such as Lactate dehydrogenase, glutathione peroxidase, and aspartate aminotransferase. Combination loaded liposomes were also able to modify DOX accumulation in the animals’ body organs which initially increased then decreased from various organs after 24 h. [66]

Another approach for combination therapy is combining a chemotherapeutic agent with other forms of therapy such as microwave ablation and radiotherapy. Wu et al. prepared DOX loaded liposomes and utilized it in combination with microwave ablation. The liposomal formulation consisted of 1,2-distearoyl-sn-glycero-3-phosphocholine (DSPC), cholesterol, and DSPE-PEG2000. Drug loaded liposomes exhibited higher cellular uptake and cytotoxicity in HepG2 and Huh7 cells compared to free drug. Applying microwave ablation enhanced the cytotoxic efficacy compared to microwave ablation alone or drug loaded liposomes alone. Combining microwave ablation with drug loaded liposomes showed the highest reduction in tumor volume and weight in HepG2 tumor bearing mice compared to the free drug, microwave ablation alone and a combination of both. Microwave ablation also possessed the ability to enhance the cellular uptake of both drug loaded liposomes and free drug both in vitro and in vivo with drug loaded liposomes showing higher uptake [67]. Shin et al. used ionizing radiation with cisplatin loaded liposomes. The prepared drug loaded liposomes was encapsulated in bio-nanocapsule expressing B virus surface antigen (HBsAg) L protein and displayed a human hepatocyte-recognizing molecule (pre-S1 peptide) possessing the ability to target human liver. The prepared liposomes composed of DPPC, cholesterol, ganglioside, diacetyl phosphate, and dipalmitoylphosphatidyl ethanolamine. The prepared drug loaded nanoparticles exhibited cytotoxicity specificity toward HCC through affecting Hep3B cells while minimally affecting human colon cancer (HCT116) cells. Applying ionizing radiation to cells pretreated with the prepared drug loaded nanoparticles exhibited the highest cytotoxicity compared to pretreatment with drug loaded liposomes and free drug. The prepared drug loaded nanoparticles had the highest reduction in tumor growth in Hep3B tumor bearing mice compared to drug loaded liposomes and free drug. Applying ionizing radiation significantly enhanced the antitumor effect of all groups in vivo with the group receiving the prepared drug loaded nanoparticles exhibiting the highest reduction. The prepared drug loaded nanoparticles were also able to mitigate the cisplatin induced nephrotoxicity [68].

Tamam et al. explored a new loading approach to increase gemcitabine entrapment entitled hypertonic loading. The new loading approach depends on admitting a high osmotic pressure which forces the water molecules including the drug through lipid bilayer. The authors prepared two gemcitabine loaded liposomes combining different loading techniques—remote loading with small volume loading (L_RS_G), and remote loading with hypertonic loading (L_RH_G)-. The liposomal formulations were composed of DPPC, cholesterol, and DSPE-PEG2000. Utilizing the different combination of loading techniques significantly enhanced the entrapment efficiency of gemcitabine compared to passive loading technique. Incorporating gemcitabine in both liposomal formulations enhanced the cellular uptake in Huh7 cells resulting in lower IC_50_ compared to free drug. L_RS_G and liposomal DOX were chosen to assess the in vivo efficacy in Huh7 tumor bearing mice. L_RS_G combined with liposomal DOX significantly delayed tumor growth time while enhancing the animals’ survival time compared to free drug combination and control group [69]. Zhang et al. prepared SOR loaded amphiphilic polypeptoids modified liposomes. Hydrophobically modified polypeptoids (HMPs) possess the ability to rupture the liposomal formulation upon hydration forming HMP-lipid fragments. These fragments possess the ability to enhance cellular uptake. SOR loaded liposomes and HMP-lipid fragments composed of L-α-phosphatidylcholine and HMPs. Blank HMP-lipid fragments were endocytosed to Huh7.5 rather than being attached to the cell wall. Blank HMP-lipid fragments were not cytotoxic. However, drug loaded HMP-lipid fragments exhibited high cytotoxicity [70].

Angiogenesis plays an important role in HCC growth since HCC is considered to be a highly vascular tumor [71]. Lian et al. explored the effectiveness of entrapping an antiangiogenic agent alongside a chemotherapeutic agent in a liposomal formulation targeting the asialoglycoprotein receptor (ASGPR). Combretastatin A4 was the antiangiogenic agent used alongside DOX. The prepared liposomes were formulated from L-α-phosphatidylcholine and cholesterol. Galactose was bound to DSPE-PEG to synthesize the targeting ligand. The formulated liposomes were successfully uptaken by both BEL7402 and Hela cells. Targeted liposomes exhibited higher cellular uptake compared to non-targeted liposomes in BEL7402. However, there was no significant difference in cellular uptake between targeted and non-targeted liposomes in Hela cells. BEL7402 and human umbilical vein endothelial (HUVEC) co-cultured system was used to mimic the interaction between tumor cells and vascular endothelial cells. Targeted liposomes showed the highest cytotoxicity and the highest anti-migration effect in comparison to blank nanoparticles, free drugs, and untargeted drug loaded nanoparticles. H22 tumor bearing mice were used to validate the results in vivo. Targeted liposomes showed higher accumulation in the tumor tissue than free DOX as well as exhibiting the highest tumor growth inhibition and the lowest tumor volume [72]. Jiang et al. also explored the co-delivery of combretastatin A4 as an antiangiogenic agent alongside a chemotherapeutic agent. The authors formulated glycyrrhetinic acid (GA) targeted liposomes containing combretastatin A4 phosphate (hydrophilic prodrug of combretastatin A4) with curcumin as the chemotherapeutic agent. The formulated liposomes composed of L-α-Phosphatidylcholine and cholesterol while the targeting ligand -GA- was bound to DSPE-PEG2000. Combination loaded liposomes was assessed in vitro against BEL7402 cells and mouse melanoma cells (B16). In vitro assessment showed higher cellular uptake for targeted liposomes in BEL7402 cells. It also showed lower cellular viability and lower migration rate when targeted, combination loaded liposomes was used in both cell lines in comparison to free drugs alone and in combination, untargeted, combination loaded liposomes and blank nanoparticles. H22 tumor-bearing mice were used to assess the formulated nanoparticles in vivo. The animal model exhibited the lowest tumor volume and the highest growth inhibition rate while confirming higher tumor uptake for the prepared liposomes [73].

Transcatheter arterial embolization utilization is hampered by the generation of hypoxia which promotes angiogenesis [74]. Zhang et al. explored the anti-cancer efficacy of curcumin loaded liposomes after transcatheter arterial embolization. Curcumin was chosen due to its previously reported anti-cancer capabilities against HCC as well as effects in suppressing hypoxic angiogenesis in HCC. Curcumin was encapsulated in liposomal formulation consisting of soybean phosphatidylcholine and cholesterol. Drug loaded liposomes exhibited higher cytotoxicity and cellular apoptosis in hypoxic HepG2 cells compared to free drug. VX2 rabbit hepatoma model was used for the in vivo evaluation. The presence of drug loaded liposomes in the embolic injection enhanced antitumor effects as well as alleviating angiogenesis compared to its absence. Drug loaded liposomes were able to decrease the expression levels of HIF-1α and surviving that were promoted by hypoxia both in vitro and in vivo. [75]

Other liposomal formulation prepared for HCC are summarized in Table 2.

**Table 2:** Summary of different liposomal formulations

Drug	Materials used	In vitro	In vivo	Summary	References
Cisplatin Curcumin	DMPC 1,2-dioleoyl-sn-glycerol-3-phosphate (DOPA) Cholesterol DSPE-PEG2000	HepG2	H22 tumor bearing mice HepG2 tumor bearing mice	High cytotoxic effect on hepG2 cells Elevated ROS levels Improved plasma retention time and antitumor effects with lower signs of toxicity in vivo	[313]
Synthetic phosphoethanolamine (amino-ethyl phosphoric ester (lipid precursor) synthesized by the research group)	Dioctadecyldimethyl-ammonium Chloride (DODAC)	Hepa1c1c7	–	Treatment was significantly more effective to promote cell death by apoptosis Elevated expression of DR4 receptor, caspases-3 and 8, cytochrome c, p53, p21, p27 and Bax Decreased expression of Bcl-2, cyclin D1, CD90 and CD44 proteins	[314]
Cantharidin	Soy phosphatidylcholine DSPE-PEG2000	HepG2	HepG2 tumor bearing mice	Enhanced cytotoxicity, apoptosis, and antiproliferative effects Improved antitumor effects in vivo	[315]
9-Nitrocamptothecin	Egg yolk phosphatidylcholine Cholesterol	HepG2 Bel-7402 Hep3B L02	HepG2 tumor bearing mice	High sensitivity of both free and drug loaded liposomes toward HepG2 cells, followed by Bel-7402, while being resistant toward Hep3B Elevated expression of p53, p21, p27, Bax, caspase-3, caspase-8, caspase-9, and mitochondrion-associated 1 Decreased expression of Bcl-2, cyclin E, cyclin A, Cdk2 and cyclin D1 Augmented antitumor effects with higher safety profile in vivo	[316]
DOX Salinomycin	HSPC Cholesterol DSPE-PEG2000	HepG2	HepG2 tumor bearing mice	High cytotoxic effect in vitro Better pharmacokinetic parameters including larger AUC, and longer t_1/2_ Enhanced tumor growth inhibition in vivo	[317]
Galactomannan	Soy phosphatidylcholine Cholesterol	–	DEN and CCL_4_ HCC model in rats	Reduced liver enzymes, PI3K/Akt, PTEN, STAT 5A gene expression, and antioxidant biomarker MDA Elevated glutathione and glutathione S-transferase levels Significant restoration of the normal hepatic architecture	[318]
Oleanolic acid	Soybean lecithin Cholesterol Triolein Stearic acid Tween 80 Polyvinyl alcohol	HepG2 H22	Pharmacokinetic and toxicity study in healthy rats H22 tumor bearing mice	High cytotoxic effect in vitro Improved pharmacokinetic parameters including longer circulation time Enhanced tumor suppression and longer survival time in vivo	[319]
Artesunate	Lecithin Cholesterol	HepG2 LO2	HepG2 tumor bearing mice	Reduced IC_50_ in vitro with almost no toxicity on normal liver cells Higher reduction in tumor growth rate and tumor weight in vivo	[320]
–	DSPC DOPE DSPE-mPEG2000 C8 PEG750 Ceramide C8 Ceramide	HepG2 SMMC-7721 Huh7 HL7702	HepG2 tumor bearing mice	High cytotoxic effect on HCC cells while showing minimal effect on normal hepatocytes Caspase dependent apoptosis, ASK1, and JNK activation while inhibiting AKT-mTOR Improved antitumor effects in vivo	[321]
di-n-butyl-di-(4-chlorobenzohydroxamato)-tin(IV) (DBDCT)	Soy phosphatidylcholine Cholesterol	–	Pharmacokinetic and tissue distribution in healthy rats H22 tumor bearing mice	Augmented pharmacokinetic parameters including larger AUC, and longer t_1/2_ Enhanced antitumor effects in vivo with higher drug concentration in RES organs and lower concentration in the adrenal glands	[322]
SOR Gadolinium	Lipoid® E80 Cholesterol	HepG2 H22	H22 tumor bearing mice	Dual acting liposomes as a therapeutic and a visualizing agent Sustained drug release leading to lower cytotoxicity than free drug Improved antitumor effects and Magnetic resonance imaging (MRI) visualization with high safety profile in vivo	[323]
–	DMPC polyoxyethylene(23) dodecyl ether	HepG2 Huh7 HC	–	High cytotoxic effect against HCC cells (high membrane fluidity) with no effect on normal hepatocytes cells (low membrane fluidity) Caspase-3 mediated apoptosis	[324]
Brucea javanica oil	Lecithin Cholesterol	HepG2	HepG2 tumor bearing mice	High cytotoxic effect in vitro Dose dependent reduction in intra-hepatic metastasis with improved survival rates in vivo	[325]
Cordycepin	Soy phosphatidylcholine Cholesterol Tween 80 Vitamin E	BEL7402 H22	H22 tumor bearing mice	Sustained drug release leading to lower cytotoxicity than free drug Dose dependent reduction in tumor growth, and tumor weight in vivo	[326]
2,6-di-isopropylphenol-linolenic acid	Egg phosphatidylcholine	HepG2	DEN HCC model in mice	The prepared drug conjugate exhibited significant cytotoxicity in vitro The prepared nanoparticles activated macrophages to engulf and suppress tumor cells Reduction in COX-2, bcl-2 expression Upregulation in Bax expression Enhanced antitumor effects with high safety profile in vivo	[327]

### Solid lipid nanoparticles

SLNs compose of lipidic core comprising solid lipids at room temperature [76] surrounded by a surfactant layer in an aqueous environment [77]. SLNs have been studied as an unconventional approach for HCC. Rahman et al. prepared SLNs composed of Compritol® ATO 888 as the solid lipid, tween 80 as the surfactant, and soy lecithin as a co-surfactant entrapping diosmin [78]. In another study, they prepared SLNs constituted of Capmul® MCM C10 as the solid lipid, poloxamer 188 as the surfactant, and soy lecithin as a co-surfactant entrapping ganoderic acid [79]. When examined on HepG2 cells, the formulated SLNs showed relatively more cytotoxicity than free drug solution and drug free SLNs. The drug loaded SLNs significantly decreased the number and size of hepatic nodules as well as enhancing the activity of endogenous antioxidants resulting in scavenging damaging free radicals in diethyl nitrosamine (DEN) animal model [78, 79].

Tunki et al. entrapped SOR in SLNs targeting ASGPR using pegylated galactose. These SLNs were formulated of glyceryl monostearate, stearic acid, soy lecithin, and tween 80. Galactosylated SOR loaded SLNs showed enhanced cellular uptake, cytotoxicity, and apoptosis in HepG2 cells in comparison to SOR alone and SOR loaded SLNs. Pharmacokinetic testing showed reduced blood clearance for galactosylated drug loaded nanoparticles while in vivo real time imaging showed targeting effect of galactosylated drug loaded SLNs [80]. The same receptor was targeted by Abd-Rabou et al. where void and drug loaded SLNs were prepared utilizing N-hexadecyl lactobionamide. The SLNs were prepared using lecithin, pluronic F68, and tween 80 and they were used to entrap either viramidine, 5-fluorouracil, or paclitaxel. In vitro testing showed higher cytotoxic effect exhibited by viramidine and paclitaxel loaded nanoparticles compared to 5-fluorouracil loaded nanoparticles as well as free drugs on HepG2 cells. Targeting specificity of the galactosylated nanoparticles was confirmed through confocal imaging as well as testing the nanoparticles on breast cancer MCF-7 cells where the SLNs showed no effect. In ovo testing was used to determine the angiogenesis index and the results showed significant reduction in the angiogenesis index using viramidine encapsulated galactosylated SLNs [81].

SLNs -like other nanoparticles- can be classified based on surface charge into anionic and cationic SLNs [82]. Surface charge plays a critical role in the delivery system stability, as well as determining the extend of nanoparticles adsorption onto the biological membranes [83]. It was reported that cationic nanoparticles enhance the NPs internalization inside the cells [84, 85]. Rahman et al. formulated SLNs entrapping resveratrol through utilizing Capmul® MCM C10 as the solid Lipid, tween 80 as the surfactant and Cetyltrimethylammonium Bromide (CTAB) as positive charge inducer. The nanoparticles showed marked increase in cytotoxicity in HepG2 cell line. In vivo analysis in DEN animal model shower higher accumulation in the tumor tissue as well as lower tumor size. It also showed lower hepatic nodule formation as well as lower expression of proinflammatory cytokines compared to free drug [86]. Chuang et al. prepared pH sensitive, pegylated cationic SLNs entrapping camptothecin in which stearylamine was used as positive charge inducer. HSPC, trimyristin, and Gelucire® 53/10 were used as the lipid core, and poloxamer 188 was used as the surfactant. The formulation was assessed against different cell lines where it inhibited the proliferation of human hepatocellular carcinoma (HCC36) and human lung carcinoma (CL1-5). Pre-clinical animal testing showed in xenograft model of the two cancers showed the ability of the SLNs to accumulate in the cancerous tissue, decrease the tumor volume while distributing quickly into the tissues [87].

However, the use of cationic SLNs is hampered by possible toxicity reports [76, 85, 88–91]. It was reported that Cationic lipids showed dose dependent toxicity which may result in hepatocellular necrosis [92]. It was also reported that cationic SLNs may decrease cell viability and show genotoxicity at concentrations that affect the cell viability [93] as well as increasing the oxidative stress of the cells [94].

Another aspect to be studied is the accumulation of nanoparticles in the tumor tissue. Only less than 10% of the administered dose is accumulated in the tumor significantly decreasing the treatment efficacy [95]. In order to further increase the SLNs accumulation in HCC cells, Varshosaz et al. assessed the usage of different sterols (cholesterol, stigmastanol or stigmasterol) to enhance SLNs containing quercetin accumulation in HepG2 cells. SLNs containing cholesterol showed the lowest IC_50_ in comparison to other sterols containing SLNs as well as free quercetin. The authors stated that this increase in membrane penetration can be attributed to higher fluency of cholesterol containing SLNs [96].

### Nanostructured lipid carriers

NLCs are considered to be second generation lipidic nanoparticles generated in order to improve the shortcomings of SLNs [97]. NLCs are modified SLNs through having the lipidic component as a mixture of solid and liquid lipids instead of solid lipid alone in SLNs [98]. The incorporation of liquid lipids transforms the perfectly crystalline structure of SLNs into imperfect amorphous structure which allows higher space for drug loading. Thus, increasing the drug loading efficacy as well as the formulation stability through reducing drug expulsion [99].

Bondì et al. entrapped SOR in NLCs composed of tripalmitin, Epikuron® 200, and Captex® 355 EP/NF or Miglyol® 812 as the lipid phase while sodium taurocholate was used as a surfactant. The formulated nanoparticles were safe to the blood erythrocytes (hemocompatibile) and did not cause cell lysis in hemolysis assay. The authors also stated that both formulations increased the bioavailability of SOR. Multiple cell lines (HepG2, Hep3B, Huh7 and PLC/PRF/5) were used to assess the cytotoxicity effect of the prepared NLCs. Drug loaded NLCs containing Captex® 355 EP/NF showed higher cytotoxicity than the free drug. Drug loaded Miglyol® 812 NLCs showed dose dependent cytotoxic effect in HepG2, Huh7 and PLC/PRF/5 cells, and no cytotoxic effects in Hep3B cells [100]. The same research group compared the ability of SLN and NLC either pegylated or non-pegylated to entrap an inhibitor of epidermal growth factor receptor (EGFR) (Tyrphostin AG-1478). The prepared nanoparticles were formulated using Compritol® 888 ATO (solid lipid), Compritol® HD5 ATO (PEGylated solid lipid), tripalmitin (solid lipid), Captex® 355EP/NF (liquid lipid), Acconon® CC-6 (PEGylated liquid lipid), Epikuron™ 200, and sodium taurocholate. Based on the nanoparticles’ characterization, un-pegylated NLCs composed of Tripalmitin, Captex® 355EP/NF, Epikuron™ 200, and sodium taurocholate was chosen as the ideal carrier for tyrphostin AG-1478 on which in vitro analysis was conducted. Drug loaded NLCs significantly inhibited colony formation ability of HA22T/VGH indicating higher cytotoxic effect [101]. Rahman et al. prepared ganoderic acid loaded NLCs which were formulated from Capmul® MCM C10, Capmul® PG8, phospholipid 90G, tween 80, and Kolliphor® P188. HepG2 exhibited lower cell viability and higher cytotoxic effect for drug loaded NLCs when compared with free drug and blank nanoparticles while showing higher cellular uptake when drug loaded NLCs were used. DEN animal model was used to validate the results in vivo. Drug loaded NLCs exhibited the lowest number of formed hepatic nodules, least levels of injury markers such as alpha fetoprotein, and alanine transaminase, significant reduction in the levels of some antioxidant markers such as glutathione and myeloperoxidase whilst elevating the levels of other antioxidant markers such as catalase and superoxide dismutase in comparison to free ganoderic acid [102].

Varshosaz et al. prepared ASGPR targeted NLCs through chemically binding lactobionic acid to stearyl amine. The prepared NLCs composed of glyceryl monostearate, lecithin, oleic acid or Labrafac®, and tween 80 or Solutol® HS15. The prepared NLCs were used to entrap 5-fluorouracil. The optimized targeted, drug loaded formula which contained oleic acid and Solutol® HS15 was assessed in vitro using HepG2 cells. The results showed that the prepared NLCs had higher cytotoxicity as well as higher cellular uptake than untargeted NLCs [103].

As mentioned earlier, HCC is considered to be one of the most resistant cancers to treatment. Liu et al. explored the ability of NLCs to overcome drug resistance through the utilization of 10-hydroxycamptothecin resistant HepG2 cells. The authors entrapped 10-hydroxycamptothecin in NLCs composed of soya oil, tween 80 and used xyloglucan as a coat to target ASGPR. The prepared NLCs were compared against microemulsion containing 10-hydroxycamptothecin and the free drug. NLCs showed the highest cytotoxicity and the highest cellular uptake. Also, in in vivo drug resistant xenograft model, NLCs showed longer residence time in the blood, higher accumulation in the liver, improved drug safety by increasing the lethal dose in comparison to free drug, as well as higher tumor inhibition rate [104]. Zhao et al. assessed the incorporation of a chemosensitizer alongside a chemotherapeutic agent using NLCs. The authors formulated NLCs composed of Precirol® ATO 5, Labrafac™ Lipophile WL 1349, Lipoid® S75, Cremophor® RH 40, and glycerin entrapping curcumin as a chemosensitizer and DOX as a chemotherapeutic agent. The cytotoxic effect of the prepared formula was validated against BEL7402 and MDR cell line BEL7402/5-FU. The results showed that the prepared NLCs containing the combination of drugs had no significant difference in cytotoxic effect on normal HCC cells compared to DOX NLCs, yet the cytotoxic effect was improved for the drug combination NLCs in MDR cell line as the NLCs caused the cell viability to become almost zero%. In DEN animal model, drug combination NLCs showed the lowest hepatic nodules, lowest liver/body weight ratio, and the lowest alanine aminotransferase and aspartate transaminase expression levels. Drug combination in NLCs also increased Caspase-3 and Bax/Bcl-2 ratio while decreasing C-myc, PCNA and VEGF [105]. Tupal et al. examined the utilization of NLCs in entrapping a pharmaceutical agent that can be used to enhance the effect of a chemotherapy. The authors encapsulated A-Tocotrienol in NLCs composed of Precirol® ATO5 as a solid lipid, Miglyol® 812 as a liquid lipid, and poloxamer 407 as a surfactant. The prepared NLCs was formulated with the aim of enhancing the efficacy of DOX. NLCs were successfully internalized in Huh7 cells as well as decreasing the anti-apoptotic protein survivin and mcl-1 mRNA expression while increasing pro-apoptotic genes Bax and Bid. On co-treatment with A-tocotrienol loaded NLCs with DOX, apoptosis of Huh7 cells was significantly enhanced showing the least cell viability percentage in comparison to free DOX, free A-tocotrienol, A-tocotrienol loaded NLCs alone and blank NLCs [106].

The process of formulating NLCs -as well as other drug delivery systems- requires the addition of pharmaceutical excipients alongside the active pharmaceutical ingredient. Excipients are defined by the International Pharmaceutical Excipients Council as “substance(s) other than the active pharmaceutical ingredient that are included in a drug delivery system to enhance the attributes of the overall safety and effectiveness of the drug delivery system during storage or use.” [107]. Excipients are generally regarded as inactive substances. Yet, new advancements in drug delivery explores the utilization of excipients in more roles other than their basic inactive effects [108]. Zhu et al. examined the utilization of coix seed oil as an active constituent for the treatment of HCC alongside its role as a liquid lipid for NLCs. The authors formulated narigin entrapped NLCs composed of glycerin monostearate as the solid lipid, coix seed oil, neodecanoate triglycerides, or oleic acid as the liquid lipid, and tween 80 as a surfactant. NLCs containing coix seed oil showed the highest cytotoxicity effect and the lower IC_50_ when tested on HepG2 cells in comparison to the free drug as and other NLCs containing other liquid lipids. NLCs containing coix seed oil also showed the highest anti-tumor effect and inhibition rate in HepG2 tumor bearing mice [109].

Another new advancement in designing drug delivery systems is the utilization of ion pair amphiphile (IPA). IPAs are cheap, lipid like alternative to phospholipids [110]. They are formed of a pair of opposite charged amphiphiles [111] that can be held together through electrostatic attraction [112]. Karmakar et al. prepared an IPA from an equimolar ratio of hexadecyltrimethylammonium bromide and sodium dodecyl sulphate. The prepared IPA was mixed with soy lecithin in different ratios to prepare NLCs. Other components used were tristearin and palmatic acid and the formulation was used to entrap oleanolic acid. Drug loaded IPA containing NLCs were compared to regular drug loaded NLCs. IPA containing NLCs showed higher stability, as well as the highest cytotoxicity and lower IC_50_ when assessed on HepG2, Huh7, and human colorectal carcinoma (HCT-116) [113].

### Microemulsion

Microemulsions are bi-continuous dispersions of oil droplets in water that are thermodynamically stable, optically isotropic. These droplets exist in a diameter larger than swollen micelles [114].

Ma et al. prepared and assessed the effectiveness of microemulsions in entrapping tanshinone [115] and tanshinone IIA [116]. The prepared microemulsions composed of ethyl oleate, phospholipid, and pluronic F68. Both microemulsion containing tanshinone and microemulsion containing tanshinone IIA were able to induce necrotic effects in H22 cells. They were also able to downregulate Bcl-2. Microemulsion containing tanshinone was able to upregulate Bax. Microemulsion containing tanshinone IIA was able to upregulate Bax as well as caspase-3. Both microemulsion formulations were able to enhance the tumor inhibition rate with smaller tumor weight than free drugs in H22 tumor bearing mice [115, 116].

Trepanier et al. incorporated CRV431 (non-immunosuppressive analogue of cyclosporine A with enhanced binding to cyclophilin) in self-microemulsifying drug delivery system. The prepared formulation consisted of vitamin E, Maisine® CC, propylene glycol, Transcutol®, and Cremophor® RH40. The prepared drug loaded nanoparticles exhibited high drug accumulation in the liver in healthy rats. The prepared drug loaded nanoparticles also exhibited higher Cmax and significantly larger AUC in healthy human subjects compared to Neoral® (the commercially available self-microemulsifying drug delivery system of Cyclosporine A) [117].

### Nanoemulsion

Nanoemulsions consist of oil, water and an emulsifier that form an emulsion with droplet size ranging from 20 to 500 nm [118]. They differ from microemulsions in being thermodynamically unstable while being kinetically stable [119].

Tabassum et al. incorporated the extract from five-day sprout of nigella sativa in nanoemulsion. Sefsol® 228, tween 80, and ethanol were used for the nanoemulsion formulation. The prepared drug loaded nanoemulsion was able to induce cytotoxicity in HepG2 while being non-toxic to normal human liver (WRL-68) cells. Treated HepG2 cells with drug loaded nanoemulsion showed increased levels of internal reactive oxygen species (ROS) which is hypothesized to be the utilized pathway for apoptosis by the prepared drug loaded nanoemulsion [120]. Nigella sativa was also explored by Usmani et al., the research group prepared self-nanoemulsifying drug delivery system co-incorporating DOX and nigella sativa oil. The prepared self-nanoemulsifying drug delivery system composed of Labrafil® M1944 CS, Kolliphor® RH40, and glycerol. The prepared drug loaded nanoparticles significantly decreased HepG2 cell viability and exhibited higher apoptotic cells compared to untreated control while being non-toxic non-malignant to Chang liver cells. The prepared drug loaded nanoparticles stimulated the production of intracellular ROS which the authors attributed to be a possible reason for the induction of apoptosis [121].

Sweed et al. formulated self-nanoemulsifying drug delivery system incorporating rosuvastatin calcium. Peceol®, tween 80, and Transcutol® P were used to construct the self-nanoemulsifying drug delivery system. Blank nanoparticles exhibited almost no toxicity in HepG2 cells indicating the safety of the prepared formulation. Drug loaded nanoparticles enhanced the cytotoxic effect of free drug in HepG2 cell as well as increased the apoptotic cells percentage [122].

Ahmad et al. prepared nanoemulsion formulation composed of Sefsol® 218, Kolliphor® RH40, and PEG400 that was used to encapsulated silymarin. Drug loaded nanoemulsion exhibited cytotoxic effect as well as apoptotic signs in HepG2 cells compared to untreated control. The prepared drug loaded nanoemulsion also promoted intracellular release of ROS to which the authors attributed the generated apoptosis. Pharmacokinetic study in healthy rats demonstrated the ability of encapsulating the drug in nanoemulsion in improving the pharmacokinetic parameters of the free drug. The prepared drug loaded nanoemulsion exhibited higher Cmax, shorter Tmax, and larger AUC compared to free drug [123].

### Phytososmes

Despite the phytochemicals robust pharmacological actions and their success in vitro. Their clinical use is limited due to their poor in vivo absorption. One of the suggested strategies to deliver phytochemicals is the usage of phytosomes [124]. Phytosomes are similar in structure to liposomes. However, they are different in the pharmaceutical agent localization. In liposomes, water soluble agents are entrapped in the inner aqueous core. In phytosomes, the active agent -partially soluble in both water and lipids- is complexed with the phospholipid head through polar and hydrogen-bonding interactions [125].

Freag et al. assessed phytosomes ability to enhance the intestinal absorption of diosmin. The research group prepared lyophilized complex of soy phosphatidylcholine and diosmin with the aim of the in vitro formation of phytosomes upon exposure to the gastrointestinal tract aqueous media. The pure drug was not able to penetrate intestinal membrane in vitro. However, drug loaded phytosomes were able to penetrate intestinal membrane and allow the drug presence in significant amount in the dissolution medium [126].

Komeil et al. prepared genistein loaded phytosomes using three different phospholipids namely Lipiod® S100, Phosal® 53 MCT, and Phosal®75 SA. The prepared phytosomes (specially Lipiod® S100 and Phosal®75 SA phytosomes) exhibited the ability to enhance the drug accumulation in the liver, blood, and intestinal serum lipoproteins in healthy rats. These results indicate the ability of the phytosomal preparation to enhance lymphatic uptake as well as protecting the drug from intestinal degradation. The prepared drug loaded phytosomes (Lipiod® S100 and Phosal®75 SA phytosomes) exhibited lower cytotoxic effect than free drug initially then had higher cytotoxic effect later on in HepG2 cells with Lipiod® S100 phytosomes exhibiting higher cytotoxic than Phosal®75 SA phytosomes. The authors attributed these results to the longer time necessary for the phytosomal preparation to exert their cytotoxic effect due to the complex structure between the drug and phospholipid. In DEN model in rats, both drug loaded Lipiod® S100 and Phosal®75 SA phytosomes exhibited higher caspase-8 expression with lower VEGF expression compared to free drug and untreated control. Drug loaded Lipiod® S100 phytosomes exhibited the highest improvement in alanine aminotransferase and aspartate aminotransferase. Drug loaded Phosal®75 SA phytosomes exhibited the highest AIF, and caspase-3 expression as well as lower MMP9 expression [127]. Loading drugs into phytosomal formulation were shown to also be hepatoprotective by Karthivashan et al. [128].

### Lipid coated nanoparticles

Coating nanoparticles can be defined as the introduction of a material on the surface of nanoparticles [129]. Shao et al. used FuGENE® HD as a lipid coat for cadmium telluride/ cadmium sulphide quantum dots. The prepared nanoparticles enhanced the cytotoxic effect of uncoated quantum dots on HepG2 cells. The prepared nanoparticles exhibited cytotoxic selectivity toward cancerous cells (Hep3B, Bel-7404, Bel-7402, SMMC-7721, Huh7, and H22) with limited cytotoxic effect on normal cells (HL-7702, CFSC-2G, H9C2, NRM, and HUVEC). The observed effect on cancerous cells was due to higher micropinocytosis dependent internalization pathways. The prepared nanoparticles exhibited high anti-tumor effect with small tumor weight compared to untreated control in H22 tumor bearing mice with no apparent toxicity resulted after administering the prepared nanoparticles [130].

Khan et al. prepared lipid coated, pH sensitive calcium carbonate nanoparticles entrapping cisplatin and oleanolic acid. The lipids used in the coating layer are HSPC, cholesterol, and DSPE-PEG2000. The prepared nanoparticles exhibited higher in vitro release in pH 5.5 compared to pH 7.4. Co-loaded nanoparticles exhibited higher cytotoxic effect compared to single drug loaded nanoparticles in HepG2 cells. Co-loaded nanoparticles were also able to ameliorate cisplatin’s induced liver toxicity resulting in higher hepatoprotection [131]. Liu et al. used galactosylceramide as a coat for hydroxycamptothecin loaded mesoporous silica nanoparticles. The used coat possesses the ability to actively target the liver. The prepared drug loaded nanoparticles were able to enhance the cytotoxicity of the free drug in HepG2 [132].

### Nanoassemblies

Nanoassemblies are core–shell structure nanoparticles that form due to the presence of a hydrophobic and hydrophilic portion co-existing together [133]. If one of the portions is inherently absent, chemical modifications can be done to render the structure amphiphilic [134]. Lipids were used to render hydrophilic moieties amphiphilic to be able to self-assemble.

Hanafy et al. prepared bromopyruvic loaded nanoassemblies composed of oleic acid conjugated chitosan. The prepared nanoassemblies were coated with folic acid conjugated bovine serum albumin in order to target folic acid receptor. Drug loaded nanoparticles exhibited higher cytotoxic effect on HLF cells compared to free drug with minimal cytotoxic effect caused by blank nanoparticles [135]. Monajati et al. conjugated cholesterol to branched polyethyleneimine forming nanoassemblies entrapping SOR. The research group formulated both pegylated and non-pegylated nanoassemblies. Drug loaded pegylated nanoparticles had higher cellular uptake than drug loaded non-pegylated nanoparticles in HepG2. The authors ascribed these results to the spherical shape of pegylated nanoparticles that possess higher uptake capability than rod shaped non-pegylated nanoparticles. Accordingly, drug loaded pegylated nanoparticles had higher cytotoxicity than drug loaded non-pegylated nanoparticles with the free drug exhibiting the highest cytotoxicity [136].

In order to increase the treatment efficacy, prodrug nanoassemblies have been developed. Prodrug nanoassemblies utilize both prodrug and nanoparticles through modifying the active constituent structure with another moiety rendering the structure amphiphilic [137]. Zuo et al. prepared 1-O-octodecyl-2-conjugated linoleoyl-sn-glycero-3-phosphatidyl gemcitabine. The prepared amphiphilic gemcitabine prodrug formed nanoassemblies either alone or in combination with cholesteryl hemisuccinate PEG 1500. The prepared prodrug possessed the ability to degrade in the presence of phospholipase A2-a highly expressed enzyme in tumor tissues-. The prepared prodrug nanoassemblies exhibited lower cytotoxicity than free drug at low concentrations. However, they exhibited higher cytotoxicity at high concentration in HepG2 cells. Combination nanoassemblies exhibited the highest tumor inhibitory rate compared to prodrug nanoassemblies and free drug in H22 tumor bearing mice. Both nanoassemblies displayed high concentrations in the liver with combination nanoassemblies exhibiting higher tumor concentration [138]. Xu et al. conjugated DOX to polylactide. The prepared conjugate formed nanoassemblies in combination with DSPE-PEG2000. To allow for active targeting, SP94 was added to the preparation as a targeting ligand. Targeted nanoassemblies exhibited higher accumulation in HCC-LM3 and BEL-7402 cells compared to non-targeted nanoassemblies. Targeted nanoassemblies were also able to specifically target HCC cells while showing no targeting capabilities toward normal liver cells (HL-7702) and lung cancer cells (NCI-H1299). Targeted nanoassemblies exhibited higher cytotoxic effect than non-targeted nanoassemblies in both cell lines. However, both nanoparticles demonstrated lower cytotoxic effect than free drug which the authors attributed to the delayed release of the drug from the prepared nanoassemblies. Targeted nanoassemblies exhibited the highest accumulation in the tumor site as well as the highest anti-tumor effects with least tumor weights and least tumor volume compared to non-targeted nanoassemblies and free drug in HCC-LM3 tumor bearing mice [139].

## Enhancing HCC uptake

**Fig. 4:**
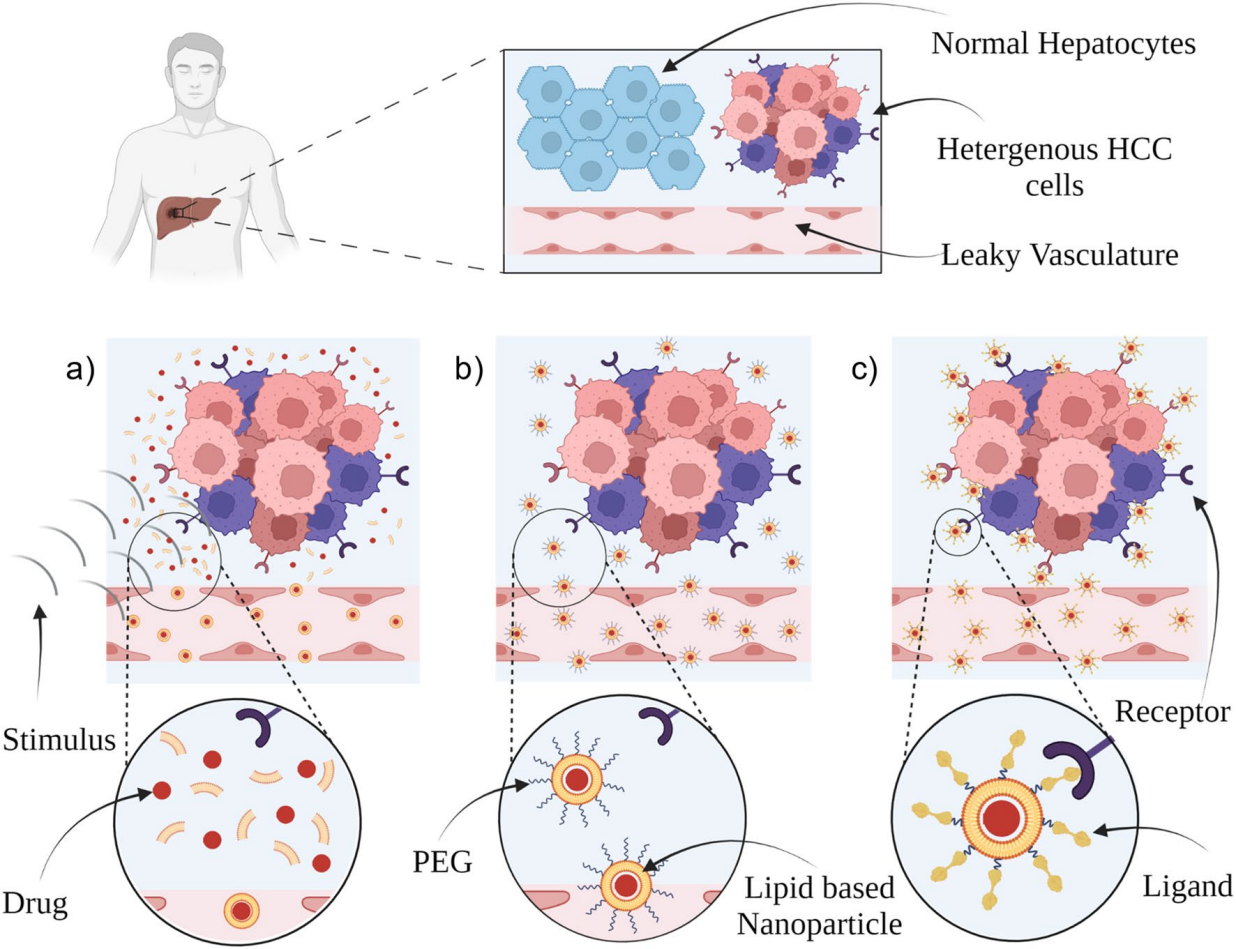
Enhancing nanoparticles uptake through exploiting the internal structure differences between healthy and diseased states through **a** Stimuli Responsive, **b** Passive Targeting, **c** Active Targeting. Created with BioRender.com

Nanoparticles face multiple biological barriers that limit their ability to greatly accumulate in the tumor tissue [40]. Upon reaching the systemic circulation, the administered nanoparticles are subjected to the first major barrier which is opsonization and subsequent uptake by mononuclear phagocyte system (MPS). Opsonins attach onto the nanoparticles surface allowing for their recognition followed by phagocytosis by the MPS [140]. The MPS consists of phagocytic cells that are predominantly located in the liver and spleen [141]. Opsonization, and hence phagocytosis, is enhanced through the presence of positive charge as well as hydrophobicity [142] and this phagocytic process significantly decreases the nanoparticles circulation in the blood stream [143]. Although MPS directs the nanoparticles to the liver which is the main site for HCC treatment, The opsonized nanoparticles will be directed to kupffer cells in the liver which results in nanoparticles elimination instead of exerting the intended pharmacological action [46, 144]. After reaching the tumor tissue, the second major barrier is the nanoparticles internalization into the cancerous cells. The cell membrane acts as a barrier for nanoparticles internalization [145]. Since nanoparticles are not able to easily penetrate the lipid bilayer of plasma membrane to be able to exert its intended actions [146]. The third major barrier after being internalized in the cells is the development of MDR. MDR results in lowering the chemotherapeutic agent concentration to sub-effective concentration which limits its efficacy [147]. Cancerous cells are characterized by the presence of efflux transporters which eject the chemotherapeutic agent out of the cancerous cells [148]. These efflux transports belong to adenosine triphosphate (ATP)-binding cassette (ABC) efflux pumps [149] which compose of 7 subfamilies that include 49 transporter proteins with varying functions [150]. Among these proteins, 3 proteins have been identified as the major contributors in cancer MDR which are P-glycoprotein (P-gp, also termed MDR1), MDR-associated protein 1 (MRP1) and breast cancer resistance protein (BCRP) [151]. P-gp can be overexpressed either due to physiological condition or as a tumor defense mechanism to the presence of chemotherapeutic agents [152]. HCC possesses high capacity to develop MDR. This drug resistance can be attributed to tumor heterogeneity and clonal evolution in response to certain pharmacological stress [153]. Tumor heterogeneity can be defined as the presence of distinct phenotypic and genetic differences among cells in the same tumor nodule or between different nodules in the same patient [154]. Primary resistance can be attributed mainly to tumor heterogeneity while acquired resistance can be attributed mainly to clonal evolution [153]. Beside the ability of lipid based nanoparticles (SLNs and NLCs) to overcome MDR in different cancers which is summarized by Lin et al., [155] various approaches have been exploited to enhance lipidic nanoparticles uptake for the treatment of HCC through targeting. The explored approaches can be classified into direct tumor delivery, stimuli responsive, passive, and active targeting (Fig. 4).

### Direct tumor delivery

Chemotherapeutic agents lack the ability to solely affect cancerous cells. This inability to discriminate between normal and cancerous cells significantly lowers the treatment efficiency [156]. To address these issues, intratumoral injection has been employed. Intratumoral injection possesses the advantage of increasing the active agent concentration in the tumor tissue while minimizing its concentration in the healthy tissues [157]. However, utilizing Intratumoral injection is hampered by its invasiveness [158]. Lipidic nanoparticles have been explored using the intratumoral injection route of administration.

Ren et al. evaluated the effect of intratumoral injection of free and liposomal DOX on the pharmacokinetics, biodistribution, and anti-tumor effect in H22 tumor bearing mice. The prepared drug loaded liposomes were able to decrease the drug concentration in the plasma and healthy organs (except for liver and spleen) while exhibiting lower toxicity and lower mortality rates. Drug loaded liposomes also significantly enhanced the tumor tissue concentration compared to the free drug. The free drug showed lower anti-tumor effect compared to drug loaded liposomes [159].

Emulsomes are a type of nanoparticles which consist of a phospholipid layer surrounding a solid lipid core [160]. Xu et al. evaluated the pharmacokinetics and biodistribution of paclitaxel loaded cationic emulsomes. HEPS tumor bearing mice were used to compare between the intravenous and intratumoral routes of administration. Plasma concertation of drug loaded nanoparticles were significantly higher using the intravenous route with much lower residence time compared to the intratumoral route. Utilizing intratumoral route significantly decreased the drug loaded nanoparticles concentration in major organs (heart, liver, spleen, lung, kidney, and pancreas) compared to intravenous route. However, intratumoral route significantly increased drug loaded nanoparticles in the tumor tissue [161].

Fu et al. prepared paclitaxel and DOX co-loaded liposomes. The prepared liposomes were loaded into thermoresponsive nanocomposite gel for intratumoral delivery. The prepared gel exhibited sol phase at 4 °C and gel phase at 37 °C with limited corrosion when incubated in phosphate buffer solution at 37 °C. The prepared liposomes showed no toxicity on three different cell lines (BEL-7402, SMMC-7221, L929) while exhibiting enhanced cellular uptake and internalization in SMMC-7221 cells. Intratumoral injection of the prepared liposomal gel significantly enhanced the accumulation in the tumor site while minimizing accumulation to other organs in SMMC-7721 tumor bearing mice. In vivo results also demonstrated the possible ability of the prepared gel to delay the payload release [162].

Irreversible electroporation is an emerging cancerous tissue ablation technique. It depends on the delivery of strong, short pulses of electric field that result in killing tumor cells as well as creating membrane defects [163]. It was also reported that the usage of electroporation significantly enhanced tumor site drug uptake [164]. Tian et al. explored the application of irreversible electroporation with intratumoral injection utilizing NVP-BEZ 235 loaded liposomes. Applying electroporation using different strengths was able to disrupt the prepared liposomes. This disruption was further validated in vitro analysis on Hep3B cells. Drug loaded liposomes exhibited significant higher cytotoxicity compared to blank liposomes. In vitro testing also demonstrated the ability of irreversible electroporation to enhance the cytotoxic effect of the prepared nanoparticles as well as free drug. Combining irreversible electroporation with the prepared liposomes improved antitumoral effect compared to its absence using intratumoral injection in Hep3B tumor bearing mice [165].

### Passive targeting

For the tumor cells to grow, excessive supply of oxygen and nutrients must be attained by the tumor cells. This need for oxygen and nutrients promote the tumor angiogenesis process through which new blood vessels supplying the tumor with its needs are formed [166]. The newly formed vasculature have poorly aligned vascular endothelia with defective structure resulting in the formation of leaky blood vessels with larger than normal pores whilst also possessing poor lymphatic clearance [167]. These vascular characteristics can be exploited by nanoparticles to passively accumulate into the tumor tissue through an effect termed enhanced permeability and retention (EPR) [168]. However, for this approach to be fully exploited, higher nanoparticles circulation time must be attained [169]. To improve nanoparticles circulation, PEG has been extensively used. PEG is used as a coat that shields the electrical charge of the nanoparticles as well as their hydrophobicity due to its electrical neutrality alongside being hydrophilic in nature [170, 171]. This shielding results in the formation of stealth nanoparticles that are not identified by the MPS which results in longer circulation time [172].

Zhao et al. prepared Fasudil loaded pegylated liposomes using DSPE-mPEG2000. The prepared liposomes showed significantly higher accumulation in the tumor tissue as well as in the liver, spleen, stomach, and kidney in Hep3B xenograft tumor model when compared to free Fasudil [173]. Huang et al. entrapped cytochalasin D in pegylated liposomes in which PEG4000 was used. Pharmacokinetic testing conducted in B16 tumor bearing mice demonstrated that pegylated liposomes released the drug more slowly and the drug existed in the blood stream for a longer time than free cytochalasin D. Tissue distribution analysis exhibited longer retention time and higher drug concentration when pegylated liposomes were used. Cytochalasin D alone was distributed in tumor tissue and other normal organs as well in high concentration specially in the liver and kidney. However, pegylated liposomes decreased the amount accumulated in the liver and kidney while having higher amount in the spleen [174]. Lin et al. utilized DSPE-PEG2000 in formulating pegylated liposomes containing berberine. Healthy nude mice were used to assess the pharmacokinetics and the effects of pegylated liposomal berberine compared to free drug. Pegylated liposomes significantly increased berberine’s half life time as well as increasing the detection time of the drug in the blood stream compared to free drug [175].

Another approach that can be used to enhance targeting is altering the composition of nanoparticles [176]. Li et al. explored the effect of changing the lipidic composition on pegylated liposomes entrapping brucine. DSPE-mPEG2000 was used to obtain pegylation and the authors assessed the effects of using Soy Phosphatidylcholine, HSPC, or a mixture between both. H22 tumor bearing mice were used to assess the differences in plasma concentration as well as drug concentration in liver, kidney, brain and in the tumor. The results showed that all liposomal formulation showed improved drug concentration when compared to free brucine, except for the concentration in the brain where soy phosphatidylcholine and mixture liposomes showed lower concentration than free drug. When comparing the three formulated pegylated liposomes, HSPC liposomes exhibited higher drug concentration in the tumor, plasma, as well as all the assessed organs. The authors attributed these results to the higher stability of HSPC pegylated liposomes since HSPC possess high T_m_ which resulted in lower drug leakage and slower drug release [177].

### Stimuli responsive

**Fig. 5:**
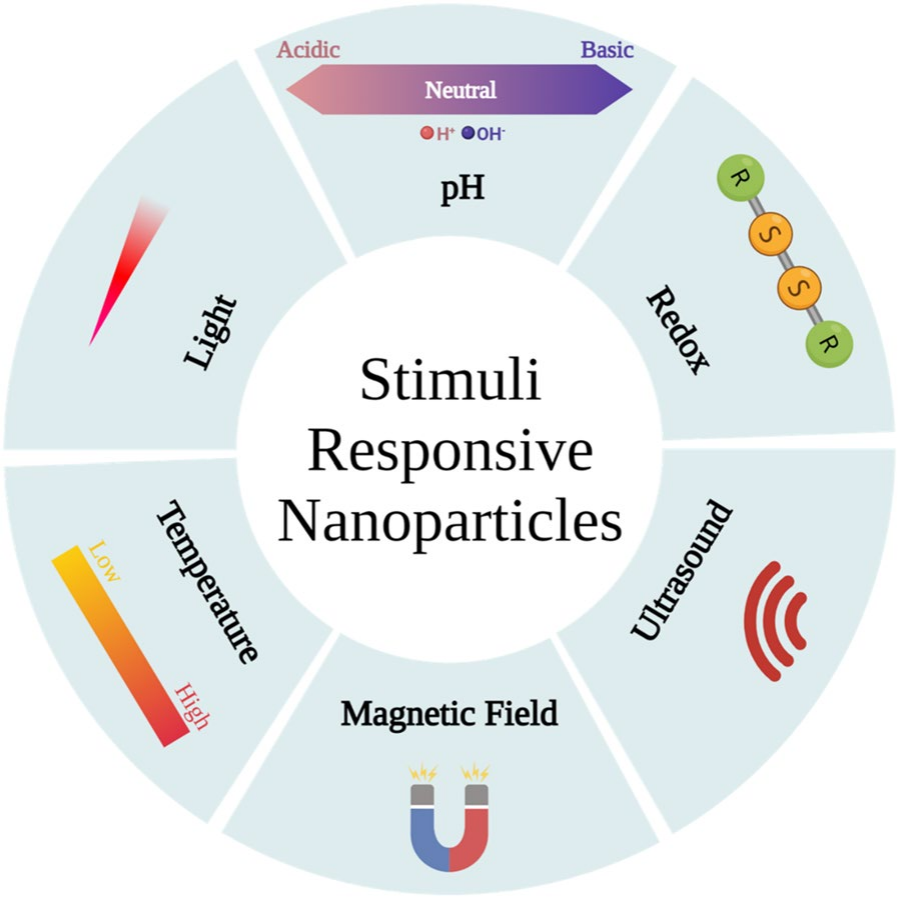
Different stimuli that affect stimuli responsive nanoparticles. Created with BioRender.com

As previously mentioned, passively targeted nanoparticles possess higher blood circulation time. However, one common limitation of this targeting approach is the possible premature release of the active payload [178]. Nanoparticles can be synthesized to respond to certain stimuli (Fig. 5). This stimulus can be either internal stimulus related to pathological changes in the cellular microenvironment or external stimulus [179]. Cancer is characterized by marked changes in the cellular microenvironment. These changes include a decrease in pH [180] and an increase in the reducing environment of the cells [181]. External stimuli include ultrasound, magnetic field, temperature, and light [182]. Several stimuli responsive lipidic nanoparticles were synthesized to enhance HCC treatment.

#### pH

pH responsive nanoparticles respond to changes in pH through two main pathways: protonation/ionization of functional groups and removal of acid-labile bonds [183]. In protonation/ionization of functional groups strategy, functional groups such as amine are added. The presence of amine group results in a shift into a positive and hydrophilic material. Amine group accepts proton and becomes hydrophilic in nature resulting in releasing the incorporated payload when the pH falls below the amine containing material pKa [184]. The second strategy depends on the incorporation of functional groups such as imine, hydrazone, and amide. These functional groups possess a pH sensitive bond that cleaves upon exposure to low pH [185].

Guo et al. prepared liposomal formulation containing DOX and MDM2 inhibitor MI-773. The liposomal formulation was coated with carboxymethyl chitosan as a pH sensitive coat. The prepared coated nanoparticles exhibited negative charge in pH 7.4 which converted into positive charge at lower pH indicating the dissociation of the carboxymethyl chitosan coat. Coated liposomes also exhibited higher in vitro drug release at pH 6.5 compared to pH 7.4. Cellular uptake in HepG2 cells was assessed in pH 6.5 and 7.4. Coated liposomes showed improved uptake in pH 6.5 compared to pH 7.4 due to the charge reversal from negative to positive [186].

Duan et al. synthesized N-acetylgalactosamine modified and pH sensitive DOX prodrug. This prodrug was co-encapsulated with SOR in lipid nanoparticles for the treatment of HCC. The prodrug gained pH sensitive capabilities due to the presence of hydrazine functional group. pH sensitive modified nanoparticles exhibited higher in vitro drug release in pH 5.5 compared to pH 7.4. The authors this increase in DOX release to the cleavage of hydrazone bonds in lower pH. They also stated that these results indicate that DOX will be released more easily in the tumor lower pH environment [187].

#### Redox

The tumor redox microenvironment is controlled by nicotinamide adenine dinucleotide phosphate (NADP) -NADPH/NADP +-and glutathione. It was reported that glutathione levels in tumor tissue is significantly higher (4 folds) than in normal tissue. Glutathione exerts its reducing effects mainly through fragmenting disulfide bonds [181]. Disulfide bonds can be incorporated using either direct or indirect method. Direct method is through the introduction of disulfide bond using disulfide containing moiety. Indirect method is through the oxidation of sulfhydryl groups [188].

Zhou et al. encapsulated DOX in redox responsive liposomal nanohybrid cerasomes through the incorporation of disulfide bond. Redox responsive nanoparticles exhibited higher in vitro release in glutathione rich release medium compared to glutathione deprived release medium. While glutathione addition showed no impact on regular nanoparticles. Redox responsive nanoparticles exhibited cytotoxic effects on SMMC-7721 cells and human breast cancer cells (MCF-7) while showing minimal effect on human embryonic kidney cells (293) [189].

#### Ultrasound

Utilizing ultrasound as a trigger for payload release is coupled with the usage of ultrasound contrast agents micro/nanobubbles. Micro/nanobubbles compose of gas-filled inner core (such as fluorocarbon) that allows the particles to oscillate at high speed when exposed to ultrasound field resulting in its destruction [190]. Employing ultrasound results in biophysical changes to the cells. When ultrasound field is used; the integrity of the cells is disrupted resulting in the formation of tiny pores that enhance the passage of micro/nanobubbles. This process is called sonoporation [191]. The second effect is cavitation. Cavitation is characterized by repetitive, yet non-destructive oscillation of micro/nanobubbles which results in enhancing the blood vessels permeability to micro/nanobubbles [192]. Employing ultrasound field also results in the induction of hyperthermia in the target tissue [193]. Micro/nanobubbles can be either polymeric or lipidic [194]. However, in this review article we will focus on lipidic micro/nanobubbles.

Zhu et al. prepared liposomal microbubble entrapping DOX using perfluoropropane as the gaseous core. Drug loaded liposomal microbubbles had a significant cytotoxic effect on H22 cells in the presence of ultrasound. However, drug loaded liposomal microbubbles had limited cytotoxicity in the absence of ultrasound. Drug loaded liposomal microbubbles enhanced the tumor ultrasound signals with high nanoparticles signals around the tumor in H22 tumor bearing mice. Tumor blood flow also increased after nanoparticles destruction indicating liposomal microbubbles’ ability to enhance blood flow inside the tumor [195].

Guo et al. used perflenapent as the gaseous core in formulating arginine-glycine-aspartic acid (RGD) modified liposomal nanobubbles loaded with fingolimod and superparamagnetic iron oxide nanoparticles. In vitro release of drug increased from the prepared liposomal nanobubbles when ultrasound was applied. The enhancement in drug releases was directly proportional to the ultrasound power applied. Cellular uptake of the prepared nanoparticles in HepG2 cells improved when ultrasound was applied compared with no ultrasound application. This improvement was attributed by the authors to the cavitation process [196].

#### Magnetic field

For nanoparticles to gain responsiveness to magnetic field, materials possessing magnetic behavior must be included. One of the most commonly used materials is iron oxide nanoparticles [197]. Utilizing magnetic field can be used to either enhance the accumulation of nanoparticles in the desired region through the application of external magnetic field, or to induce hyperthermia [198]. The induced hyperthermia can be used either as a trigger for payload release from thermosensitive nanoparticles [199], or as a direct heat source for tumor cells ablation [200]. Lipidic nanoparticles incorporating iron oxide nanoparticles will be focused upon.

Grillone et al. prepared SOR and superparamagnetic iron oxide nanoparticles loaded solid lipid nanoparticles. The prepared solid lipid nanoparticles enhanced cellular uptake in HepG2 cells when magnetic field was applied. Magnetic field application was able to focus the cytotoxic effect in the desired tumor tissue as a result of magnetically driven tumor accumulation [201].

Chen et al. encapsulated DOX with superparamagnetic iron oxide nanoparticles in PEG stabilized liposomes. The in vitro release of the entrapped drug significantly increased upon the application of magnetic field. Drug loaded nanoparticles cytotoxicity significantly improved upon applying magnetic field compared to its absence in Huh7 cells [202].

Liu et al. formulated DOX loaded thermosensitive liposomes. Radiofrequency responsive manganese and zinc doped superparamagnetic iron oxide nanoparticles were used in mixture with the liposomal formulation. The combination was used to induce hyperthermia allowing the release of the encapsulated payload. The authors utilized a strong static gating field containing a sharp zero point superimposed on the radiofrequency field. This modification allowed radiofrequency waves to affect only nanoparticles at zero or near zero point. Thus, increasing the resolution of the targeted release. In vitro release of DOX increased with increasing the temperature of the dissolution medium. DOX in vitro release also increased with the presence of magnetic nanoparticles. Liposomal formulation in combination with magnetic nanoparticles exhibited higher cytotoxic effect in Huh7 cells in the presence of magnetic field compared to its absence [203].

#### Temperature

Increasing the tumor tissue temperature to 40–43 °C is termed hyperthermia [204]. As mentioned earlier, inducing hyperthermia possess the advantages of direct tumor cells killing as well as triggering release from thermosensitive nanoparticles. Inducing hyperthermia is also capable of enhancing the nanoparticles uptake in the tumor tissue through increasing tumor vascular permeability, alongside improving local blood flow at the heated area [205]. Triggering payload from thermosensitive nanoparticles using hyperthermia attracted significant attention due to its ability to control the payload release through steering the heating focus and heating power [206].

Direct tumor killing using microwave ablation by heat generation was explored by Zhou et al.. Sodium chloride that is used as a thermo-seed was encapsulated in liposomal formulation. Liposomes loaded with sodium chloride exhibited better heat conversion than blank liposomes and free sodium chloride. Sodium chloride loaded liposomes exhibited the highest cytotoxic effect on HepG2 cells while showing hemocompatibility on the application of microwave. The in vivo efficacy of the prepared liposomes was assessed in both HepG2 xenograft animal model and MHHC97H orthotropic animal model. In both models, the prepared nanoparticles exhibited the highest anti-tumor effect while applying microwave compared to the usage of free sodium chloride, microwave ablation alone, and the prepared nanoparticles alone without applying microwave [207].

Guo et al. prepared icaritin loaded microemulsion with coix seed oil as an unconventional liquid phase. The prepared microemulsion was entrapped in thermosensitive liposomes. In vitro release of the entrapped drug was slow at 37 °C. However, in vitro release was significantly higher at 42 °C. The prepared microemulsion and thermosensitive liposomes exhibited enhanced cellular uptake in HepG2 cells compared to free drug using clathrin-mediated internalization pathway. Applying mild hyperthermia enhanced the prepared nanoparticles cytotoxicity. The prepared liposomes enhanced tumor uptake and exhibited higher tumor penetration in HepG2 + LX-2 desmoplastic 3D tumor spheroids before hyperthermia application. Hyperthermia application enhanced the in vivo anti-tumor effect of the thermosensitive liposomes compared to the anti-tumor effect in the absence of hyperthermia in HepG2 + LX-2 tumor bearing mice [208].

Zhu et al. modified iron oxide nanoparticles with oleic acid. These modified magnetic particles were encapsulated alongside hydroxycamptothecin to obtain thermosensitive magnetic liposomes. Drug in vitro release was enhanced when the release media was heated to 42 °C compared 37 °C confirming the thermosensitive characters of the prepared nanoparticles. The prepared drug loaded nanoparticles enhanced tumor treatment in Huh7 tumor bearing mice when magnetic strips and external heating source (42 °C water bath) were applied compared to the anti-tumor effect in their absence [209].

Multiple clinical trials were conducted validating the efficacy of DOX loaded thermosensitive liposomes as an adjuvant therapy to radiofrequency ablation [210, 211].

#### Light

Phototherapy is described as the utilization of light (preferably near-infrared light) through activating phototherapeutic agents. These agents should possess low toxicity in the dark as well as the ability to kill cancer cells under light activation without affecting normal cells [212]. Phototherapy processes can be classified into photothermal therapy, photodynamic therapy, and triggered release of encapsulated payload in nanoparticles [213]. Photothermal therapy depends on the generation of thermal energy upon exposure to light. The generated heat directly kills cancerous cells [214]. Photodynamic therapy relies on generating a specific ROS called singlet oxygen (^1^O_2_) upon activating a photosensitizer. The generated ^1^O_2_ is responsible for killing cancer cells [215]. Payload release upon light activation depends on co-loading the intended pharmaceutical agent with a photothermal agent in thermosensitive nanoparticle. Upon light activation, the encapsulated photothermal agent raises the nanoparticles temperature allowing the triggered release of the payload [216].

Youssef et al. encapsulated hypericin in solid lipid nanoparticles. The encapsulated photosensitizer exhibited lower degradation when compared to free photosensitizer. Both free and encapsulated photosensitizer exhibited no significant effect on HepG2 cells in the dark. However, upon light exposure, both free and encapsulated photosensitizer exhibited significant cytotoxicity. The free photosensitizer exhibited higher cytotoxic effect. These results were attributed by the authors to the possible quenching deactivation of the photosensitizer as a result of the solid lipid nanoparticles compact and thick structure [217].

A new photosensitizer -thiophenyl sulfonated zinc phthalocyanine ((PhS.SO_3_Na)_4_ZnPc)- was synthesized by Abdel Fadeel et al.. The prepared photosensitizer was evaluated for its photodynamic efficacy after being loaded into liposomes and transferosomes [218]. Transferosomes are a modified form of liposomes with the addition of an edge activator. This addition allows the formation of flexible, ultra-deformable vesicle [219]. The research group utilized HepG2 cells to validate the cytotoxic effects in vitro. Encapsulating thiophenyl sulfonated zinc phthalocyanine in both nanoparticles enhanced its cytotoxicity compared to its free form with liposomes exhibiting the highest cytotoxicity. Applying light further enhanced the cytotoxic effect compared to its absence [218].

Pradhan et al. prepared gold coated photothermal liposomes entrapping quercetin. The prepared liposomes were thermosensitive allowing the release of quercetin upon raising the temperature after light exposure. In vitro release of the encapsulated drug significantly increased upon raising the temperature from 37 °C to 45 °C. The prepared nanoparticles exhibited varying cytotoxic effect in different cell lines upon light exposure. Huh7 cells had the highest cytotoxicity followed by HeLa cells then B16F10. Drug loaded nanoparticles exhibited higher cytotoxic effect than void nanoparticles upon light exposure at the same time and intensity. The obtained results also showed that the prepared nanoparticles both drug loaded and void possess good hemocompatibility [220].

Photothermal, Photodynamic, and triggered drug release therapies were co-assessed by He et al.. The research group co-encapsulated SOR and indocyanine green (photosensitizer and photothermal agent) in thermosensitive liposomes. Encapsulating the photosensitive agent in liposomal formulation enhanced its photostability compared to free agent. The drug in vitro release from the prepared thermosensitive liposomes increases significantly upon light application. The prepared nanoparticles were able to attain more cellular uptake in Hep3B cells upon light activation. This enhancement in cellular uptake was attributed by the authors to the production of ROS and the generation of thermal effect which promoted cellular uptake. The cytotoxic effect of the prepared nanoparticles on Hep3B cells significantly improved upon light activation compared to the cytotoxic effect in absence of light. The prepared nanoparticles also had higher anti-tumor effect in Hep3B tumor bearing mice upon light activation compared to its absence [221].

One of the potential risks of phototherapy is the induction of severe hypoxia as a result of oxygen consumption in generating ROS. This may result in a rise in cancer metastatic risk [222]. Yang et al. formulated light-activatable liposomes entrapping tetravalent platinum prodrug (Pt(IV)) and chlorin e6 (Ce6) -photodynamic-. Incorporating Pt(IV) consumes glutathione for its transfer to Pt(II). Depleting glutathione can alleviate the generated hypoxia. The prepared liposomes contained unsaturated phospholipids possessing the ability to transform to hydrophilic peroxides by ROS. This transformation allows for the payload release. In vitro release of incorporated payload improved significantly upon light exposure. The prepared nanoparticles were able to enhance cellular uptake of the incorporated payload in both cisplatin sensitive liver cells (7404) and cisplatin resistant lung cancer cells (A549DDP) before the application of light. Cytotoxicity of the prepared nanoparticles improved significantly upon light activation in 7404, A549DDP, cisplatin sensitive lung cancer (A549), and cisplatin resistant liver cancer (7404DDP). The prepared nanoparticles also enhanced tumor site accumulation in PDHC tumor bearing mice. Upon light activation the payload released in the tumor site was higher than in the absence of light [223].

### Active targeting

**Fig. 6:**
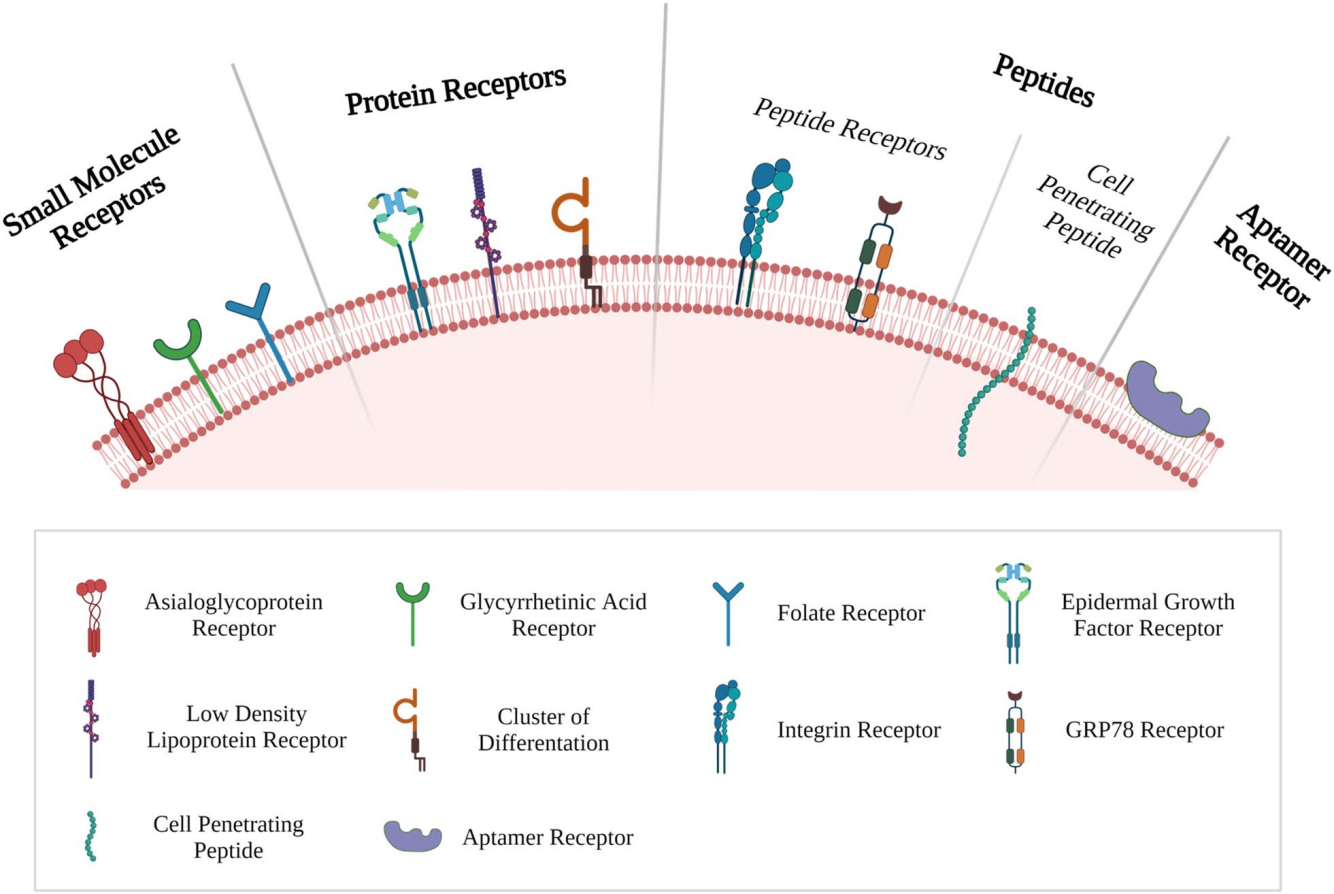
Different receptors targeted by lipidic nanoparticles through active targeting. Created with BioRender.com

Higher understating of disease progression and molecular targets has allowed the development of nanoparticles that actively target the tumor tissue on a cellular level limiting nanoparticles unspecific binding to healthy organs and tissues [40]. Active targeting utilizes a ligand that specifically binds to an overexpressed receptor on the tumor surface allowing for receptor mediated endocytosis and higher drug uptake [224]. As previously mentioned, nanoparticles that reach systemic circulation are directed to kupffer cells in the liver resulting in an un-intended uptake by these cells. Utilizing ligands in active targeting possess the advantage of directing the decorated nanoparticles into the intended cancerous cells while minimizing un-intended uptake by normal cells [225]. Higher drug uptake and increased drug localization can result in enhancing the treatment efficacy [226]. Active targeting also can enhance the treatment efficacy through by-passing P-gp drug efflux mechanism [227]. Despite the fact that utilizing active targeting is more difficult than passive targeting due to multiple factors including additional chemical synthesis, and quality control steps, active targeting has been extensively explored in enhancing lipidic nanoparticles uptake for HCC treatment [228]. Multiple receptors have been utilized by lipidic nanoparticles to enhance their uptake in HCC treatment (Fig. 6). These receptors can be classified into small molecule receptors, protein receptors, peptide receptors, and aptamer receptors [46].

#### Small molecule receptors

##### Asialoglycoprotein receptor

ASGPR is a C-type lectin composed of 48 kDa (ASGPR-1) and 40 kDa subunits (ASGPR-2) that is responsible for clearing glycoproteins that terminate with a galactose or N-acetylgalactosamine moiety [229]. ASGPR is highly expressed in hepatocytes with very low expression in other locations in the body where it internalizes materials into hepatocytes using clathrin enabled receptor-mediated endocytosis [230]. ASGPR has been heavily studied in targeting HCC using various moieties including galactose or N-acetylgalactosamine.

Pathak et al. utilized arabinogalactan conjugated to cholesterol as the targeting moiety for DOX loaded liposomes. Non-targeted drug loaded liposomes showed comparable cell inhibition on HepG2, breast cancer (MCF7), colon cancer (HT-29), and lung cancer (A549) cells. While targeted liposomes showed higher uptake in HepG2 cells. In vivo biodistribution was conducted on healthy mice through radiolabeling the formulated nanoparticles. Both liposomal formulations were able to accumulate in the liver and spleen with minor amounts being present in the bladder. These results indicate the ability of both nanoparticles to mitigate DOX mediated nephrotoxicity. In vivo analysis in HepG2 tumor bearing mice exhibited smaller tumor volume when compared to free drug [231]. Bansal et al. developed targeted liposomes entrapping oxaliplatin using lactobionic acid coupled to DSPE. BEL7402 cells demonstrated higher cellular uptake for targeted liposomes compared to non-targeted liposomes. Targeted liposomes exhibited higher accumulation in tumor and liver and lower concentration in spleen and kidney compared to non-targeted liposomes in MDA-MB-231 tumor bearing mice [232].

In a research done by Ding et al., the effectiveness of three different ligands was assessed in enhancing cellular uptake of 10-Hydroxycamptothecine loaded liposomes. Galactose and di-galactose were bound to stearic acid targeting ASPGR while galactose and biotin were co-bound to stearic acid targeting both ASPGR and biotin receptors. Galactose-biotin liposomes showed the highest cellular uptake in HepG2 cells followed by di-galactose liposomes then galactose liposomes. Cellular uptake studies also exhibited low uptake in A549 (lung cancer cells), Hela (cervical carcinoma cells), and SGC-7901 (gastric cancer cells). All targeted liposomes also exhibited lower cellular uptake in LO2 (normal hepatocytes) than HepG2 cells [233].

Qu et al. synthesized Octanoyl galactose ester as a targeting ligand for HCC which was used to provide active targeting capabilities to formulated microemulsion. Targeted microemulsions showed higher uptake than non-targeted microemulsion in HepG2 cells. Cellular uptake studies also showed that targeted microemulsion uptake decreased significantly when HepG2 cells were pre-treated with galactose. Targeted microemulsion exhibited higher localization in tumor site with longer retention time when compared to non-targeted microemulsion in HepG2 tumor bearing mice. The authors also stated that there was no difference in pharmacokinetics between orally administered targeted and non-targeted microemulsion which the authors attributed to the lack of effect on galactosylation modification on oral absorption [234].

Wei et al. utilized both passive and active targeting approaches in enhancing the delivery of DOX. DSPE-PEG2000 was used to convert the liposomes into pegylated liposomes as well as conjugating the targeting moeity which was Lactoferrin. Targeted liposomes showed higher cellular uptake in ASPGR positive HCC cell lines HepG2, BEL7402, and SMMC7721. However, both targeted and non-targeted liposomes showed no difference in cellular uptake in ASPGR negative mouse embryonic fibroblast cell line NIH 3T3. DOX showed the highest cellular uptake in all cell lines which the authors attributed to DOX ease of diffusion into the cells while nanoparticles accumulate in the cell using receptor mediated and non-receptor mediated endocytosis [235].

The possibility to tackle both MDR as well as active targeting to HCC using mitoxantrone loaded liposomes was explored by Zhang et al.. The research group synthesized galactosyl conjugated Pluronic P123 utilizing P123 ability to reverse MDR while targeting ASPGR using the galactosylated moiety. The ability of P123 to reverse MDR was validated using BCRP-overexpressing Madine-Darby Canine kidney cell line. P123 exhibited dose dependent enhancement in the drug accumulation while galactosylated P123 showed comparable effect indicating that galactosylation possesses limited effect on MDR reversal. Targeted liposomes and P123 modified liposomes showed comparable enhancement in mitoxantrone uptake when compared to conventional liposomes. Conventional liposomes also improved mitoxantrone uptake compared to free drug which the authors attributed to the ability of liposomes to bypass efflux transporters. Targeted liposomes showed higher cellular uptake in ASPGR positive Huh7 cells when compared to P123 modified liposomes and conventional liposomes. Liposomal formulations were able to improve free drug pharmacokinetics with targeted liposomes showing the highest AUC and longest T_1/2_. Huh7 orthotopic tumor xenograft model in mice as well showed higher accumulation for targeted liposomes in the tumor area [236].

A summary of other approaches targeting ASPGR is provided in Table 3.

**Table 3:** Summary of different approaches targeting ASGPR

Targeting Ligand (Conjugated Moiety)	Drug	Materials used	Nanocarrier	In vitro	In vivo	References
Galactose (DSPE-PEG2000)	Celastrol	Soy phosphatidylcholine Cholesterol	Liposomes	HepG2	AKT/c-Met induced HCC mouse model	[328]
Butyryl galactose ester	–	Coix seed oil Coixan Cremophor® RH40 PEG400	Microemulsion	HepG2	HepG2 tumor bearing mice	[329]
Stearyl galactose	–	Coix seed oil Coix seed polysaccharide Cremophor® RH40 PEG400	Microemulsion	HepG2 L02 Caco-2	HepG2 tumor bearing mice	[330]
Galactose (Pluronic P123)	Irinotecan	(2E)-4-(dioleostearin)-amino-4-carbonyl-2-butenonic (DC) [pH sensitive lipid] Phospholipid PC-98 T CTAB Tetraethyl Orthosilicate	Lipid coated mesoporous silica nanoparticles	Huh7 L02	Pharmacokinetics in healthy mice Huh7 ectopic and orthotopic tumor xenograft model in mice	[331]
Lactoferrin (DSPE-PEG2000-COOH)	–	Soy phosphatidylcholine Cholesterol DSPE-PEG2000	Liposomes	HepG2 NIH 3T3 ECV304	Pharmacokinetics in healthy mice HepG2 tumor bearing mice	[332]
Lactoferrin (DSPE-PEG2000-Mal)	Triiodothyronine (T3)	1-palmitoyl-2-oleoyl-glycero-3-phosphocholine (POPC) Dimethyldioctadecyl-ammonium -bromide salt- (DDAB) DSPE-PEG2000	Liposomes	FaO HepG2 SKHep	–	[333]
Asialofetuin (DSPE-PEG2000-Mal)	–	DSPC Cholesterol DSPE DSPE-PEG2000	Liposomes	HepG2	Tissue distribution in healthy rats	[334]
Lactobionic Acid (1,2-dioleoyl-sn-glycero-3-phosphoethanolamine (DOPE))	DOX	Egg phosphatidylcholine Cholesterol DSPE-mPEG	Liposomes	HepG2	Pharmacokinetics in healthy mice HepG2 tumor bearing mice	[335]
Arabinogalactan (Palmitate)	DOX	Lipoid® S 100 Lipoid® S PC 3 Cholesterol	Liposomes	HepG2 MCF7 A549 HT29	Pharmacokinetics in healthy rats Tissue distribution in healthy mice HepG2 tumor bearing mice	[336]
Lactose (DOPE)	-	DMPC 1,2-Dimyristoyl-sn-glycero-3-phosphoglycerol (DMPG) 1,2-distearoyl-3-trimethylammonium-propane chloride (DSTAP) lauric acid coated magnetite nanocores	Magnetoliposomes	HepG2 C17.2	-	[337]

##### Glycyrrhetinic acid receptor

GA is the aglycon derivative of the widely present in licorice roots glycyrrhizic acid [237]. GA is of significant importance since it is the bioavailable form in the body as the carbohydrate moiety of glycyrrhizic acid is removed by the actions of intestinal bacterial [238]. HCC is characterized by the presence of both glycyrrhetinic acid receptor (GAR) and glycyrrhizic acid receptor. However, GAR binding sites are much more than glycyrrhizic acid receptors binding sites [239] resulting in the exploration of GAR as a target for HCC.

**Fig. 7:**
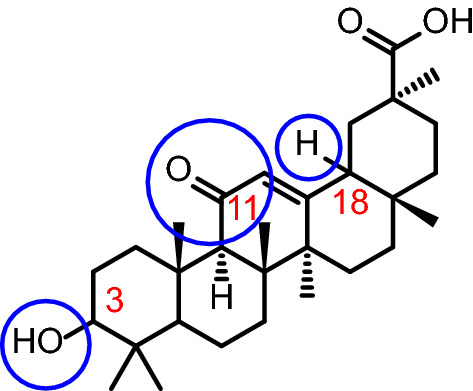
Summary of glycyrrhetinic acid modifications done by Sun et al.. [241]

Cellular uptake and clearance mechanism of both free GA and GA modified liposomes in HepG2 cells were evaluated by Sun et al.. Free GA showed both concentration and temperature dependent uptake with higher uptake achieved in higher concentrations and higher temperature. Different proteins showed varying effects on GA uptake. Bovine serum albumin showed no interference in GA uptake. Fetal bovine serum and cytoplasm protein slightly decreased GA uptake while cytomembrane protein showed the highest decrease in GA uptake. GA coupled liposomes showed time dependent uptake with higher uptake achieved with longer incubation time. GA coupled liposomes showed higher dependence on active transport, endocytosis, and caveolae-dependent endocytosis cellular uptake mechanisms while clathrin-dependent endocytosis and micropinocytosis were not significantly utilized by GA coupled liposomes. Free GA clearance best fitted exponential decay kinetics while GA coupled liposomes best fitted second order kinetics [240]. The same research group assessed the efficacy of different GA derivatives (18β-GA, 18α-GA, 3-acetyl-18β-GA, and 11-deoxy-18β-GA) in targeting HCC. The synthesized ligands were assessed in vitro using HepG2 cells both alone and bound to liposomal formulations through conjugation with DSPE-PEG2000. 18β-GA and 3-acetyl-18β-GA showed the highest targeting ability through significantly reducing the ability of GA to bind to HepG2 cells. 18α-GA showed the least effect with 11-deoxy-18β-GA showing moderate targeting effect. The results indicate the importance of 18-H configuration in the targeting efficiency with the β configuration more effective than the α configuration. The results also indicated the ability to enhance the targeting effect through the addition of 3-acetyl as well as the removal of the 11-carbonyl group of GA (Fig. 7). 18β-GA and 3-acetyl-18β-GA liposomal formulations showed the highest affinity to HepG2 while all liposomal formulation showed enhanced targeting ability with increasing the incubation time. H22 tumor bearing mice were used the targeting ability in vivo for the liposomal formulations. 18β-GA and 3-acetyl-18β-GA liposomal formulations showed the highest localization in the tumor tissue while all liposomal formulations showed comparable accumulation in the liver [241].

Chu et al. coupled GA to DSPE-PEG2000 and used it as a targeting moiety for curcumin loaded NLCs. Varying concentrations of GA were used to assess whether cellular uptake increases with increasing the targeting ligand concentration on HepG2 cells. The results showed that with increasing the ligand concentration, the cellular uptake increases until reaching a certain limit then decreases. The authors attributed these results to the overabundance of ligands which may result in stearic hinderance which may result in blocking ligand-receptor interactions. Targeted NLCs with the best ligand concentration exhibited higher cellular uptake in HepG2 in comparison to free drug, conventional NLCs, and pegylated NLCs [242].

Docetaxel loaded liposomes were modified by Li et al. through synthesizing 3-succinyl-30-stearyl GA. Non-targeted liposomes showed no difference in cellular uptake in L02 cells and hepatic nonparenchymal cells (LX-2) while targeted liposomes showed enhanced uptake in L02 cells through receptor mediated endocytosis. Targeted liposomes had enhanced pharmacokinetic profile compared to non-targeted liposomes when assessed in healthy rats. However, the difference was not significant between the two groups. The authors attributed these results to the inability of hydrophilic succinic anhydride to overcome the hydrophobicity of GA. This led to rapid MPS uptake of hydrophobic targeted and non-targeted liposomes [243].

Zhou et al. explored the potential of synthesizing a single ligand that has the ability to target two receptors simultaneously. The authors synthesized 3-Galactosidase-30-stearyl deoxyglycyrrhetinic acid (11-DGA-3-O-Gal) which has the ability to target both GAR and ASPGR. The synthesized ligand was used to modify cantharidin loaded liposomes. In vivo testing in healthy rats exhibited lower T_1/2_ and higher elimination rate for targeted liposomes which the authors ascribed to the targeted liposomes’ ability to distribute quickly to the tissues as well as quick recognition by GAR and ASPGR. Targeted liposomes were able to significantly enhance liver targeting compared to non-targeted liposomes. The results also demonstrated that targeted liposomes accumulated mainly in the liver and kidney with higher amount in the kidney [244]. Double active targeting was also explored by Li et al. in modifying DOX loaded liposomes. GA and peanut agglutinin were conjugated to DSPE-PEG2000 and used to bind to GAR and β-D-galactosyl-(1–3)-N-acetyl-D-galactosamine that forms mucin 1 protein respectively. In vitro validation was done using MUC1-negative HCC cell line HepG2, MUC1-positive HCC cell line SMMC-7721, and MUC1-positive breast cancer cell line MCF-7. GA modified liposomes and peanut agglutinin modified liposomes showed the highest cellular uptake in HepG2 cells and MCF-7 cells respectively. Dual ligand liposomes showed the highest uptake in SMMC-7721 utilizing caveolae-mediated endocytosis and micropinocytosis internalization mechanisms. All liposomal formulations (non-targeted, GA modified, peanut agglutinin modified, and dual ligand liposomes) were able to enhance tumor accumulation with dual ligand liposomes exhibited the highest tumor uptake in SMMC-7721 tumor bearing mice [245].

##### Folate Receptor

Folate Receptor (FR) is a family of proteins that are classified into FRα, FRβ, FRγ and FRδ. They are a group of glycoproteins where FRα, FRβ, and FRδ are glycosyl-phosphatidylinositol anchored cell surface proteins while FRγ is a secreted protein with no anchor [246]. Folate receptors are characterized by their expression in multiple tumors but very low expression in normal tissues. Among the different isoforms of FR, FRα have been identified as the most common isoform [247]. FRα is widely expressed in multiple tumors such as breast, pancreatic, and non-small cell lung cancer [248]. However, FRβ is the isoform present in liver cancer through existing in tumor associated macrophages that have been linked with promoting metastasis [249]. Although liver cancer does not usually show high levels of folate receptors [250], targeting folate receptors have been explored in targeting lipidic nanoparticles.

Folic acid was conjugated to DSPE-PEG2000 by Liu et al. and they used this conjugation to modify diacid metabolite of norcantharidin loaded liposomes. Biodistribution study in H22 tumor bearing mice demonstrated high accumulation of targeted and pegylated liposomes in the tumor, liver, and spleen. This accumulation was significantly higher than the free drug accumulation. Targeted liposomes were able to further enhance pegylated liposomes’ ability to target the tumor. However, targeted liposomes showed significantly higher accumulation in the kidney compared to pegylated liposomes. The authors stated that this higher accumulation in the kidney requires further safety evaluation for targeted liposomes containing diacid metabolite of norcantharidin due to higher risk of kidney toxicity [251]. Liu et al. as well explored the enhancement of diacid metabolite of norcantharidin using folate receptor. Diacid metabolite of norcantharidin was co encapsulated with ABT-737 in lipid coated chlorodimethyloctadecylsilane mesoporous silica nanoparticles modified by folic acid conjugated DSPE-PEG2000. Targeted nanoparticles accumulated in H22 cells significantly higher than AML12 cells compared to non-targeted nanoparticles [252].

Wang et al. prepared docetaxel lipid based nanosuspensions modified using folic acid conjugated DSPE-PEG2000. Targeted and pegylated nanosuspensions showed comparable cytotoxic effect on FR negative HepG2 cells. However, targeted nanosuspensions showed significantly more cytotoxic effect than pegylated nanosuspensions on FR positive B16 cells. Both nanosuspensions improved the cytotoxic effect of free drug on both cell lines. Both targeted and pegylated nanosuspension improved the pharmacokinetic parameters of free drug with higher residence time, and T_1/2_ with lower clearance in B16 tumor-bearing mice. Targeted nanosuspensions improved tumor targeting efficiency compared to free drug while pegylated nanosuspensions showed intermediate improvement in targeting efficiency. Both nanosuspensions were accumulated in the liver and spleen more than free drug while decreasing the free drug concentration in the heart, lung, and kindey [253].

#### Protein receptors

##### Epidermal growth factor receptor

EGFR is a single chain transmembrane glycoprotein [254]. EGFR (ErbB1/ HER1) belongs to ErbB family of tyrosine kinase surface receptors which includes as well ErbB2 (HER2/neu), ErbB3 (HER3) and ErbB4 (HER4) [255, 256]. EGFR plays an important role in cell proliferation, apoptosis, angiogenesis, and metastasis [257]. HCC is characterized by the overexpression of EGFR [258] which led to its exploration as a possible target for drug delivery using lipidic nanoparticles.

EGFR was targeted by lipidic nanoparticles using EGFR peptide [259], or EGFR antibody [260]. Lin et al. decorated the surface of paclitaxel loaded magnetic polymeric liposomes with EGFR peptide (NH2-YHWYGYTPQNVI-GGGSGGGS-Cys-COOH). EGFR modification enhanced the cellular internalization of the prepared nanoparticles compared to non-modified nanoparticles in SMMC-772 cells. In vivo analysis exhibited higher drug concentrations in tumor site when EGFR peptide modified nanoparticles were used alongside the application of external magnetic field when compared to free drug and modified nanoparticles without magnetism [259].

Gao et al. prepared lipid polymer hybrid nanoparticles entrapping adriamycin and conjugated EGFR Fab´ to DSPE-PEG-maleimide(Mal) to be used as the targeting moiety. EGFR Fab´ conjugated nanoparticles showed higher cellular uptake when compared to non-modified nanoparticles. This improvement in cellular internalization was observed in SMMC-7721 (high expression of EGFR), HepG2 (moderate expression of EGFR) and Huh7 (low expression of EGFR) cells. However, the enhancement in cellular uptake in Huh7 was moderate. EGFR Fab´ conjugated nanoparticles enhanced tumor tissue uptake in SMMC-7721 tumor bearing mice when compared to non-modified nanoparticles. Non-modified nanoparticles exhibited low cellular internalization in the tumor tissue. However, EGFR Fab´ conjugated nanoparticles demonstrated high cellular internalization in a pattern consistent with receptor mediated endocytosis [260].

##### Low density lipoprotein receptor

Low density lipoprotein receptor (LDLR) composes of several domains, most importantly class A repeat and class B repeat. Class A repeat consists of three pairs of cysteines in each seven or eight repeat moieties. Each seven or eight repeat moieties consist of approximately 40 amino acids. Class B repeat contains four-amino-acid sequence of Tyr-Trp-Thr-Asp. Domains containing class B repeat typically consist of both class B repeats and epidermal growth factor repeats. The class B repeats form a structure called β-propeller [261]. Among LDLR proteins, LDLR1 is reported to be involved in cancer progression with its specific role in tumor invasion and migration depending on the tumor type. Regarding HCC, LDLR1 shows higher expression in non-recurrent HCC compared to early recurrent HCC. Higher LDLR1 expression also was correlated to lower metastatic potential [262].

Apo B was explored as a targeting ligand for LDLR by Wang et al.. The research group utilized Apo B as a targeting ligand for lipid nanoparticles entrapping both SOR and dihydroartemisinin. The prepared nanoparticles were able to enhance the cellular uptake in LDLR + ve HepG2 cells compared to non-targeted nanoparticles. This enhancement in uptake was mediated through receptor mediated endocytosis [263].

Alanazi et al. explored the ability to target LDLR conjugating cholesterol to an active pharmaceutical ingredient. This exploration was based on the theoretical knowledge that this conjugation will result in a cholesterol ester which is a natural component of LDLR. The research group conjugated 5-fluorouracil to cholesterol and incorporated this conjugation into both liposomes and LDL nanoparticles. Drug conjugation to cholesterol was able to enhance drug partitioning into LDL core due to its hydrophobic nature compared to non-conjugated drug. When comparing conjugated and non-conjugated drug loaded liposomes in healthy rats, conjugated drug loaded liposomes significantly enhanced the drug accumulation in liver. Conjugated drug loaded liposomes also were able to accumulate in LDL while non-conjugated drug loaded liposomes failed to accumulate in LDL [264].

LDL nanoparticles packed with docosahexaenoic acid targeting LDLR were explored by both Ou et al. [265] and Yang et al. [266]. Ou et al. explored the mechanistic pathway of LDL nanoparticles induction of cell death in HCC. Human liver tumor cell lines (PLC/PRF/5 and HepG2) as well as rat hepatoma cell line (H4IIE) were used for the in vitro assessment while HepG2 tumor bearing mice was the chosen model for the in vivo evaluation. The results demonstrated that the LDL nanoparticles packed with docosahexaenoic acid induce cell death through ferroptosis. Upon nanoparticles cellular internalization, nanoparticles result in the depletion of cellular stores of glutathione. The activity of GPX4 is impeded as a result of the depletion of glutathione stores as well as an unknown mechanism exerted by the nanoparticles. The downregulation of GPX4 as well as a large influx of the nanoparticles lead to an increase in lipid peroxidation. This cascade of events lead to the induction of ferroptosis cell death [265]. Yang et al. assessed the effects of LDL nanoparticles packed with docosahexaenoic acid on cancer stem cells isolated from human hepatoma cell lines. Epithelial cellular adhesion molecule (EpCAM) positive and cluster of differentiation (CD)133 negative (EpCAM + CD133 −) cancer stem cells as well as EpCAM negative and CD133 negative (EpCAM − CD133 −) adult cancer cells were derived from two human HCC cell lines (Huh7 and HepG2). Both cells derived from both Huh7 and HepG2 showed no significant difference in their LDLR expression. The prepared nanoparticles induced apoptosis through enhancing lipid peroxidation and increasing ROS levels. In vivo testing was conducted directly inoculating H4IIE cells (rat HCC cell line) into rats’ liver. The results of in vivo testing demonstrated that EpCAM + CD133 − cancer stem cells are more resistant to treatment using LDL nanoparticles packed with docosahexaenoic acid compared to EpCAM − CD133 − adult cancer cells [266].

##### Cluster of differentiation

CD are cell surface glycoprotein that are used to immunophenotype cells. There are more than 350 CD markers that exhibit various functions including cell adhesion, cell activation, and cell inhibition [267]. Multiple CD were exploited using lipidic nanoparticles to target HCC.

CD13 (also called aminopeptidase N) protein is a 150 kDa metalloprotease characterized by the presence of a catalytic domain oriented toward the extracellular matrix. It plays a vital role in multiple processes including cytokines activity regulation through cleaving the present N-terminals as well as regulating inflammatory mediators [268]. Pang et al. functionalized docetaxel loaded lipid based nanosuspensions using asparagine-glycine-arginine (NGR) peptide. Pegylated and NGR modified nanosuspensions were able to enhance cellular uptake compared to regular nanosuspensions in HepG2. Regular, pegylated and NGR modified nanosuspensions showed higher accumulation in HepG2 cells compared to human normal liver cells (HL-7702). NGR modification greatly enhanced nanosuspension accumulation in the tumor tissue compared to pegylated and regular nanosuspensions in H22 tumor bearing mice [269].

CD90 (also called Thy-1) protein is 25 to 37 kDa glycophosphatidylinositol anchored protein with heavy N-glycosylation on two sites in human and three sites in mouse. CD90 exerts roles in cell–cell and cell–matrix interactions [270]. Yang et al. utilized CD90 antibody as a targeting moiety for thermosensitive magnetoliposomes. CD90 + ve liver cancer stem cells were separated from Huh7 cell line and used for in vitro assessment against normal Huh7 cells and CD90-ve Huh7 cells. CD90 targeted nanoparticles exhibited the highest cellular uptake in CD90 + ve liver cancer stem cells compared to non-targeted nanoparticles and CD20 targeted nanoparticles. CD90 targeted nanoparticles slightly enhanced cellular uptake in normal Huh7 cells while showing no improvement in CD90-ve Huh7 cells [271].

CD147 (also called basigin) protein is a type-1 transmembrane glycoprotein that exerts functions in intercellular recognition. CD147 can be present in either a 27 kDa unglycosylated form or a 43 to 66 kDa glycosylated form [272, 273]. Wang et al. formulated DOX loaded liposomes and used metuximab (bivalent fragment HAb18F(ab’)2 derived from CD147-specific monoclonal antibody) as a targeting moiety. Huh7 cells were chosen for subsequent analysis since Huh7 cells demonstrated the highest expression of CD147. In vitro testing demonstrated enhanced cellular uptake when using targeted liposomes compared to non-targeted liposomes with the free drug exhibiting the highest cellular uptake. Both targeted and non-targeted liposomes were able to improve the AUC as well as decrease clearance, V_d_, and t_1/2_ compared to free drug in healthy rats. Huh7 tumor bearing mice demonstrated higher ability for targeted liposomes to accumulate in the tumor as well as liver and spleen with lower accumulation in heart, lung, and kidney. In vivo evaluation also confirmed the ability of targeted liposomes to enhance tumor uptake compared to non-targeted liposomes and free drug [274].

#### Peptides

Peptides are low molecular weight protein fragments that compose of two or more amino acids linked together using peptide (amide) bond [275]. Peptides possess the advantages of low immunogenicity, in vivo integrity, and easy conjugation techniques [276]. Employing peptides is done through either binding to peptide receptors or using cell penetrating peptides. Cell penetrating peptides possess the ability to penetrate cells through bypassing cellular uptake barriers and to reach the cell’s nucleus [277, 278].

##### RGD

RGD is a peptide that has the ability to bind to integrin receptors [279] which are responsible for regulating cell–cell and cell-extracellular matrix interactions. Among the various integrin receptor isoforms, αvβ3 plays a significant role in tumor angiogenesis through over expression on tumor vasculature and other tumor cells with limited expression on normal vasculature [280]. Integrin receptors especially αvβ3 are overexpressed in HCC [281] which led to their exploration as a targeting ligand with lipidic nanoparticles.

Wang et al. formulated SOR and quercetin loaded lipid coated PLGA nanoparticles and used RGD conjugated DSPE-PEG as the targeting moiety. Targeted nanoparticles exhibited higher cellular uptake in HepG2 cells when compared to non-targeted combination loaded nanoparticles, non-targeted SOR loaded nanoparticles, and free drugs both the combination and SOR alone [282].

RGD conjugated DSPE-PEG was used by Fei et al. to modify arsenic trioxide loaded liposomal shell-mesoporous silica core hybrid nanoparticles. In vitro study was conducted in αvβ3 positive HepG2 and αvβ3 negative MCF-7 and L02. Targeted nanoparticles exhibited significantly more uptake in HepG2 than MCF-7 and L02. Non-liposome shell mesoporous silica nanoparticles showed lower uptake due to the nanoparticles aggregation on cell surface impeding their uptake. Targeted and non-targeted nanoparticles exhibited enhanced bioavailability and prolonged residence time than non-liposome shell mesoporous silica nanoparticles and free drug due to lower drug leakage in healthy rats. H22 tumor bearing mice showed higher accumulation in tumor tissue by all nanoparticles. Targeted nanoparticles exhibited the highest accumulation in the tumor tissue [283].

iRGD peptide (CRGDK/RGPDC) is a combination between RGD sequence motif and cryptic CendR motif [284]. This sequence combination allows iRGD to possess both tumor targeting properties and neuropilin-1 -dependent tissue-penetrating properties through proteolytic exposure of its C-terminal end [285]. Zhang et al. modified DOX and SOR co-loaded lipid-polymer hybrid nanoparticles using iRGD conjugated DSPE-PEG-Mal. Targeted nanoparticles showed enhanced cellular uptake in αvβ3 positive HepG2 with no significant difference in cellular uptake between targeted and non-targeted nanoparticles in αvβ3 negative L02 cells. Pharmacokinetics testing in healthy rats demonstrated improvements in bioavailability and blood retention time of DOX and SOR when both targeted and non-targeted nanoparticles were used. Targeted nanoparticles exhibited highest anti-tumor activity in HepG2 tumor bearing mice as a result of activation of Caspase-3 pathway of cell apoptosis and mitochondria-mediated cell death [286].

##### SP94

SP94 (SFSIIHTPILPL) is an HCC targeting peptide that was identified using phage display technique [287]. It was reported that the molecular target to which SP94 binds to has not been identified yet [288]. However, A recently published report identified GRP78 receptor to be the molecular target of SP94 [289]. GRP78 receptor is a 78 kDa glucose regulated protein that belongs to the heat-shock protein 70 (HSP70) family. GRP78 receptor exerts effects in controlling protein folding, preserve protein stability, as well as effects in inducing apoptosis [290]. The receptor shows high expression on HCC cells in the majority of patients while not being expressed on normal hepatocytes [291].

Wu et al. enhanced targeting ability of DOX and vinorelbine co-loaded liposomes using SP94 conjugated to DSPE-PEG-N-hydroxysuccinimide(NHS). The ability of SP94 to enhance targeting was explored using DOX alone as the model drug. Targeted liposomes showed improved cellular uptake in SK-HEP‑1 cells when compared to pegylated liposomes. Targeted liposomes exhibited higher tumor accumulation while showing similar uptake to pegylated liposomes in normal organs in Mahlavu tumor bearing mice [292].

The effectiveness of SP94 modification was compared to the effectiveness of galactose modification in enhancing the targeting effect by Jiang et al.. SP94 or galactose was conjugated to DSPE-PEG2000 to modify C14 alkyl chain norcantharimide derivative loaded liposomes. Both galactose and SP94 modified liposomes were able to enhance cellular uptake in HepG2 cells compared to conventional liposomes. However, SP94 modification showed completely different uptake pattern than galactose modified liposomes. SP94 modified liposomes were able to internalize within the cells at a much faster rate than galactose modified liposomes and then decline over time. While galactose modified liposomes showed increased accumulation over time. The authors ascribed SP94 faster uptake to its possible ability to allow the ligands to bind to multiple binding sites through the enhancement of clustering and mobility of ligands. This effect is attributed to the SP94 binding to peg-terminal “brush” on the fluid lipid membrane. SP94 modified liposomes were able to enhance drug concentration in the tumor tissue while decreasing its accumulation in normal organs in H22 tumor bearing mice [293].

To assess the efficacy of different peptides in targeting HCC, Wu et al. conjugated L-peptide, SP94 peptide, and PC5-52 peptide to liposomal iron oxide nanoparticles and DOX loaded liposomes. L-peptide, which is an anti-cancer cell membrane, SP94 peptide, and PC5-52-peptide, which is anti-tumor endothelia. L-peptide and SP94 showed comparable cellular uptake in HepG2 and Huh7. Cellular uptake increased when combining the two peptides. L-peptide was also able to avoid binding to normal hepatocytes, same as SP94. Drug loaded liposomes showed some toxicity signs in the liver, kidney, and spleen. However, L-peptide or SP94 or both peptides modified drug loaded liposomes showed no signs of toxicity in HepG2 tumor bearing mice. The in vivo model also demonstrated the ability to enhance the chemotherapeutic effect of DOX when PC5-52-peptide modified drug loaded liposomes is co-administered with either L-peptide or SP94 modified drug loaded liposomes. The authors concluded from the in vivo results that combining anti-tumor and anti-endothelial peptide is more effective than a combination of two anti-tumor peptides [294].

##### TAT

TAT (GRKKRRQRRRPPQ) is the transcriptional activator protein in HIV-1. It is a cationic cell penetrating peptide which consists of arginine and lysine residues [295]. TAT -like other cell penetrating peptides- lacks target specificity which hinders their clinical application [296]. This led to combining TAT with other targeting approaches for the treatment of HCC using lipidic nanoparticles.

Mei et al. formulated multistage liposomes composed of cleavable PEG, RGD, and TAT. Long chain cleavable PEG was employed as the first stage to achieve passive targeting. The second stage composed of RGD conjugated to medium chain PEG to recognize and bind to HCC cells. The third and inner stage is TAT conjugated to short chain PEG to enhance cellular internalization. Cellular uptake was assessed using HeLa cells (low expression of integrin receptors) and HepG2 cells (high expression of integrin receptors). RGD modification improved cellular uptake in HepG2 compared to HeLa cells. TAT co-modification showed synergistic effect in improving cellular uptake in HepG2 cells. Cysteine addition to cells (allow the PEG to detach from the liposomes) exhibited higher cellular uptake for cleavable PEG liposomes suggesting that the removal of the outer PEG layer exposed the targeting ligands and enhanced cellular uptake. Multistage liposomes exhibited high dependence on clathrin-dependent uptake pathway. HepG2 tumor bearing mice demonstrated the ability of PEG coating to allow the liposomes to evade the reticuloendothelial system (RES). The in vivo model exhibited higher ability of multistage liposomes to internalize into the tumor tissue with higher stability [297].

Zhao et al. prepared phase-transformation lipid nanoparticles entrapping 10-hydroxycamptothecin and coated with liquid perfluoropentane. The prepared nanoparticles possess the ability to transform into lipid microbubbles upon exposure to external ultrasound radiation with a specific intensity. The prepared nanoparticles were modified using cysteine flanked TAT (CG-TAT-GC) to enhance cellular internalization as well as hyaluronic acid to add target cellular specificity through binding to CD44. Double modified nanoparticles were able to adhere to SMMC-7721 cells which overexpress CD44. However, cysteine flanked TAT modified nanoparticles were not able to adhere to SMMC-7721 cells. SMMC-7721 tumor spheroid showed the ability of the double modified nanoparticles to penetrate the 3D tumor. Yet, hyaluronic acid modified nanoparticles were not able to penetrate the 3D tumor efficiently. SMMC-7721 tumor bearing mice were used to assess the targeting ability in vivo. Double targeted nanoparticles exhibited higher accumulation in the tumor site when compared to non-targeted nanoparticles as well as cysteine flanked TAT modified nanoparticles [298].

#### Aptamer receptors

Aptamers are defined as nucleic acid molecules that have the ability to bind to specific targets through folding into complex 3D structures that mimic antibodies [299]. Ding et al. used liver cancer-specific aptamer TLS11a as a targeting moiety. The research group prepared DOX loaded into TAT modified mesoporous silica nanoparticle incorporated within aptamer bearing liposomes. Aptamer and TAT co-modified nanoparticles and aptamer modified nanoparticles were able to localize the prepared nanoparticles in the nucleus of H22 cells. This localization was higher in case of aptamer and TAT co-modified nanoparticles with TAT modified nanoparticles showing the least cellular uptake. Aptamer and TAT co-modified nanoparticles and TAT modified nanoparticles significantly enhanced accumulation in tumor tissue in H22 tumor bearing mice with the higher improvement is exhibited using the aptamer and TAT co-modified nanoparticles [300].

Other less explored active targeting approaches using lipidic nanoparticles are summarized in Table 4.

**Table 4:** Summary of less utilized targeting techniques for HCC

Targeting Ligand (Conjugated Moiety)	Receptor (Receptor Classification)	Drug	Materials used	Nanocarrier	In vitro	In vivo	References
Cholic Acid (DSPE-PEG)	Cholic acid transporters (Small molecule receptors)	DOX Silybin	Lipoid® Cholesterol	Liposomes	HepG2 HCC97H H9c2 H22	Pharmacokinetics and tissue distribution in healthy mice H22 tumor bearing mice HepG2 tumor bearing mice	[338]
Fucoidan (DSPE-PEG2000-Amine)	P-selectin receptor (Small molecule receptors)	DOX 1-butyl-3-methylimidazolium-L-lactate (microwave sensitizer)	DPPC Cholesterol	Liposomes	HepG2 H22	H22 tumor bearing mice	[339]
Lysine (Polyoxyethylene 40 stearate)	Amino acid transporter ATB^0,+^ -also called SLC6A14- (Small molecule receptors)	Docetaxel	Soybean phospholipids Cholesterol	Liposomes	HepG2 H22 L929	H22 tumor bearing mice	[340]
AMD3100	CXCR4 receptor (Small molecule receptors)	SOR	DOPA d-α-tocopherol polyethylene glycol 1000 succinate (TPGS) PLGA	Lipid coated PLGA nanoparticles	HCA-1 JHH-7	orthotopic HCA-1 tumors in mice	[341]
NK4 (DSPE-PEG2000-Mal)	c-Met receptor (Protein receptors)	10-Hydroxy-camptothecin	Soybean phospholipids Cholesterol	Liposomes	HepG2	Pharmacokinetics in healthy rats Tissue distribution in healthy mice	[342]
^D^T7 peptide—retro inverse analogue of ^L^T7 peptide- (DSPE-PEG2000)	Transferrin receptor -also called CD71 [46]- (Protein receptors)	Docetaxel	HSPC Cholesterol DSPE-PEG2000	Liposomes	HepG2	Pharmacokinetics in healthy mice HepG2 tumor bearing mice	[343]
Carbonic anhydrase IX antibody (DSPE-PEG2000-Mal) BR2 peptide (DSPE-PEG2000-Mal)	Carbonic anhydrase IX receptor (Protein receptors) Cell penetrating peptide [344] (Peptides)	Cantharidin	Soybean lecithin DSPE-PEG2000	Liposomes	HepG2	Orthotopic HepG2 tumor model in mice	[345]
BR2 peptide (DSPE-PEG2000-Mal)	Cell penetrating peptide (Peptides)	Cantharidin	Soybean lecithin DSPE-PEG2000	Liposomes	HepG2	HepG2 tumor bearing mice	[346]
Octreotide peptide (Polyethylene glycol 100 monostearate)	Somatostatin receptor (Peptides)	Hydroxy-camptothecine	Soybean phospholipids Trilaurin Labrafac® CC Vitamin E Glycerol	Nanostructured Lipid Carriers	L02 SMMC-7721	Pharmacokinetics in healthy rats	[347]
Glutathione (DOPE)	γ-glutamyltrans-peptidase (Peptides)	DOX	HSPC Cholesterol	Liposomes	Huh7 HUVEC ECDHCC NIH/3T3 BxPC3	Huh7 tumor bearing mice BxPC3 tumor bearing mice	[348]
G12 peptide (DSPE-PEG2000-Mal)	Glypican-3 (Peptides)	SOR IR780 iodide	Egg phosphatidyl-choline Cholesterol	Liposomes	HepG2 HL-7702 H22	HepG2 tumor bearing mice H22 tumor bearing mice	[349]
GPC3 peptide (DSPE-PEG2000-biotin)	Glypican-3 (Peptides)	Apatinib	DSPC DSPE-PEG2000 Perfluoro-propane	Nanobubble (ultrasound destructible)	HepG2	-	[350]

## Conclusion and future perspective

HCC is considered to be one of the most challenging diseases worldwide with less-than-optimal treatment outcomes using chemotherapeutic agents. Lipidic nanoparticles gained significant attention due to their stability, biocompatibility, and their ability to decrease undesirable side effects. Utilizing lipidic nanoparticles significantly enhances the cytotoxic activity of the used anti-neoplastic agents. Further enhancements in the efficiency of lipid based nanoparticles can be achieved through various targeting techniques. Targeting approaches drastically enhance the tumor uptake of the intended anti-neoplastic agents while minimizing the effect on normal tissues. Lipid based nanoparticles hold great promise in improving the treatment outcomes of anti-neoplastic agents for HCC. However, further improvements are required to achieve higher number of clinically approved medications. A special focus should be directed toward decreasing the possible risk of toxicity. Alternatives to the possibly toxic cationic lipids as well as solutions to achieve the encouraging effects of positively charged lipidic nanoparticles without the associated toxicity risk should be attained to further enhance the safety profile of the prepared formulations. Another area of focus to further increase bench to bedside translation is the methods of preparation used. Novel, green, and ecofriendly methods of preparation that do not depend on organic solvents will further improve the scalability of production and decrease production costs while eliminating the risk of the presence of toxic residuals.

## Data Availability

Not applicable.

## Abbreviations

11-DGA-3-O-Gal: 3-Galactosidase-30-stearyl deoxyglycyrrhetinic acid
^1^O_2_: Singlet Oxygen
AASLD: American Association for the Study of Liver Diseases
ABC: ATP Binding Cassette
ASGPR: Asialoglycoprotein Receptor
ATP: Adenosine Triphosphate
BCLC: Barcelona Clinic Liver Cancer
BCRP: Breast cancer resistance protein
CD: Cluster of Differentiation
Ce6: Chlorin e6
CLIP: Cancer of the Liver Italian Program
CTAB: Cetyltrimethylammonium Bromide
DDAB: Dimethyldioctadecylammonium -Bromide Salt-
DEN: Diethyl Nitrosamine
DMPC: 1,2-Dimyristoyl-sn-glycero-3-phosphocholine
DMPG: 1,2-Dimyristoyl-sn-glycero-3-phosphoglycerol
DODAC: Dioctadecyldimethylammonium Chloride
DOPA: 1,2-Dioleoyl-sn-glycerol-3-phosphate
DOPC: 1,2-Dioleoyl-sn-glycero-3-phosphocholine
DOPE: 1,2-Dioleoyl-sn-glycero-3-phosphoethanolamine
DOX: Doxorubicin
DPPC: 1,2-Dipalmitoyl-sn-glycero-3-phosphocholine
DSPC: 1,2-Distearoyl-sn-glycero-3-phosphocholine
DSPE: 1,2-Distearoyl-sn-glycero-3-phosphoethanolamine
DSTAP: 1,2-Distearoyl-3-trimethylammonium-propane chloride
EASL: European Association for the Study of the Liver
EGFR: Epidermal Growth Factor Receptor
EpCAM: Epithelial Cellular Adhesion Molecule
EPR: Enhanced Permeability and Retention
FR: Folate Receptor
GA: Glycyrrhetinic Acid
GAR: Glycyrrhetinic Acid Receptor
HCC: Hepatocellular Carcinoma
HKLC: Hong Kong Liver Cancer
HMPs: Hydrophobically Modified Polypeptoids
HSP70: Heat-shock protein 70
HSPC: Hydrogenated Soy Phosphatidylcholine
IPA: Ion Pair Amphiphile
LDLR: Low Density Lipoprotein Receptor
Mal: Maleimide
MDA: Malondialdehyde
MDR: Multidrug Resistance
mPEG: Methoxy(polyethylene glycol)
MPS: Mononuclear Phagocyte System
MRI: Magnetic Resonance Imaging
MRP1: Multidrug resistance associated protein 1
NADP: Nicotinamide Adenine Dinucleotide Phosphate
NGR: Asparagine-glycine-arginine
NHS: N-hydroxysuccinimide
NLCs: Nanostructured Lipid Carriers
PEG: Polyethylene Glycol
P-gp: P-glycoprotein
PLGA: Poly Lactic-co-Glycolic Acid
POPC: 1-Palmitoyl-2-oleoyl-glycero-3-phosphocholine
Pt: Platinum prodrug
RES: Reticuloendothelial System
RGD: Arginine-glycine-aspartic acid
ROS: Reactive Oxygen Species
SLNs: Solid Lipid Nanoparticles
SOR: Sorafenib
T3: Triiodothyronine
TACE: Transarterial Chemoembolization
TARE: Transarterial Radioembolization
TPGS: D-α-tocopherol polyethylene glycol 1000 succinate
VEGFR: Vascular Endothelial Growth Factor Receptor
WHO: World Health Organization
